# The putative tumour suppressor protein Latexin is secreted by prostate luminal cells and is downregulated in malignancy

**DOI:** 10.1038/s41598-019-41379-8

**Published:** 2019-03-26

**Authors:** Robert I. Seed, Alberto J. Taurozzi, Daniel J. Wilcock, Giovanna Nappo, Holger H. H. Erb, Martin L. Read, Mark Gurney, Leanne K. Archer, Saburo Ito, Martin G. Rumsby, John L. Petrie, Aled Clayton, Norman J. Maitland, Anne T. Collins

**Affiliations:** 1 Cancer research Unit, Department of Biology, University of York, Heslington, York YO10 5DD UK; 2 Department of Biology, University of York, Heslington, York YO10 5DD UK; 3 Institute of Metabolism and Systems Research, College of Medical and Dental Sciences, University of Birmingham, Edgbaston, Birmingham B15 2TT UK; 4 Tissue Microenvironment Group, Division of Cancer and Genetics, Tenovus Building, Heath Park, Cardiff University CF14 4XN, Cardiff, UK; 5 Division of Infection and Immunity, School of Medicine, Cardiff University, Heath Park, Cardiff, CF14 4XN UK; 6 Department of Pathology, University of California San Francisco, San Francisco, CA 94110 USA

## Abstract

Loss of latexin (LXN) expression negatively correlates with the prognosis of several human cancers. Despite association with numerous processes including haematopoietic stem cell (HSC) fate, inflammation and tumour suppression, a clearly defined biological role for LXN is still lacking. Therefore, we sought to understand LXN expression and function in the normal and malignant prostate to assess its potential as a therapeutic target. Our data demonstrate that LXN is highly expressed in normal prostate luminal cells but downregulated in high Gleason grade cancers. LXN protein is both cytosolic and secreted by prostate cells and expression is directly and potently upregulated by all-trans retinoic acid (atRA). Whilst overexpression of LXN in prostate epithelial basal cells did not affect cell fate, LXN overexpression in the luminal cancer line LNCaP reduced plating efficiency. Transcriptome analysis revealed that LXN overexpression had no direct effects on gene expression but had significant indirect effects on important genes involved in both retinoid metabolism and IFN-associated inflammatory responses. These data highlight a potential role for LXN in retinoid signaling and inflammatory pathways. Investigating the effects of LXN on immune cell function in the tumour microenvironment (TME) may reveal how observed intratumoural loss of LXN affects the prognosis of many adenocarcinomas.

## Introduction

Prostate cancer (CaP) is now the most prevalent non-cutaneous cancer in men^1^. Despite the success of surgical intervention, radiotherapy and androgen ablation therapies, there remains a significant recurrence rate of up to 30%^2,3^. Such recurrences almost inevitably lead to more aggressive and treatment-resistant cancer such as castrate resistant and neuroendocrine tumours^4^. Therefore, by improving our knowledge of the human prostate it could be possible to devise new rational therapeutics which target the unmet clinical need of treatment resistant disease.

Latexin (LXN) was first discovered in the lateral neocortex of rats and serves as a marker of neuronal development^5^. More recently LXN has been identified as a quantitative trait gene responsible for the negative regulation of haematopoietic stem cells (HSC) in mice^6,7^. Other studies suggest that increased LXN expression is associated with inflammatory responses such as acute pancreatitis and inflammatory lung disease^8^. In mouse models, LXN is found to be both highly expressed in inflammatory Mast cells and is induced following stimulation of mouse macrophages^9,10^

Several additional studies have also linked loss of LXN expression with numerous human malignancies, such as leukeamia^11^, melanoma^12^, hepatocellular carcinoma^13^ and pancreatic ductal adenocarcinoma (PDAC)^14^. For example, LXN expression was shown to significantly correlate with tumour size, histological grade, metastasis and clinical stage in PDAC, indicating that LXN may function as a tumour suppressor^15^. Despite its association with numerous malignancies, the normal function of LXN and subsequently its role in malignant progression remain enigmatic. Initially, LXN was reported to function as the sole endogenous Carboxypeptidase A4 (CPA4) inhibitor in mammals^16^, but more recent studies have shown that LXN can function independently of CPA4^11,17^. Furthermore, in different cancer models the biological effects of LXN appear to be diverse and its biological function is increasingly multi-faceted.

The expression and function of LXN in the normal and malignant prostate also remain poorly characterised. A single publication to date demonstrates that LXN might be a retinoic acid responsive gene and impart tumour suppressive effects on CaP cells. In this study, transient knockdown of LXN in non-malignant cells resulted in increased cell motility, invasiveness and clonogenic capacity, and preliminary data in prostate epithelial cell lines suggested that transient overexpression of LXN may reverse these effects^18^. Such data highlight LXN as a potentially interesting candidate for prospective therapeutic targeting of treatment resistant CaP.

Therefore, we aimed to further characterise the expression patterns and define a molecular function for LXN in the normal and malignant prostate in order to determine how potential loss of LXN signalling in advanced prostate cancer might be exploited as a rational therapeutic target in CaP.

## LXN is highly expressed in prostate luminal cells but is downregulated in malignancy

**Figure 1:**
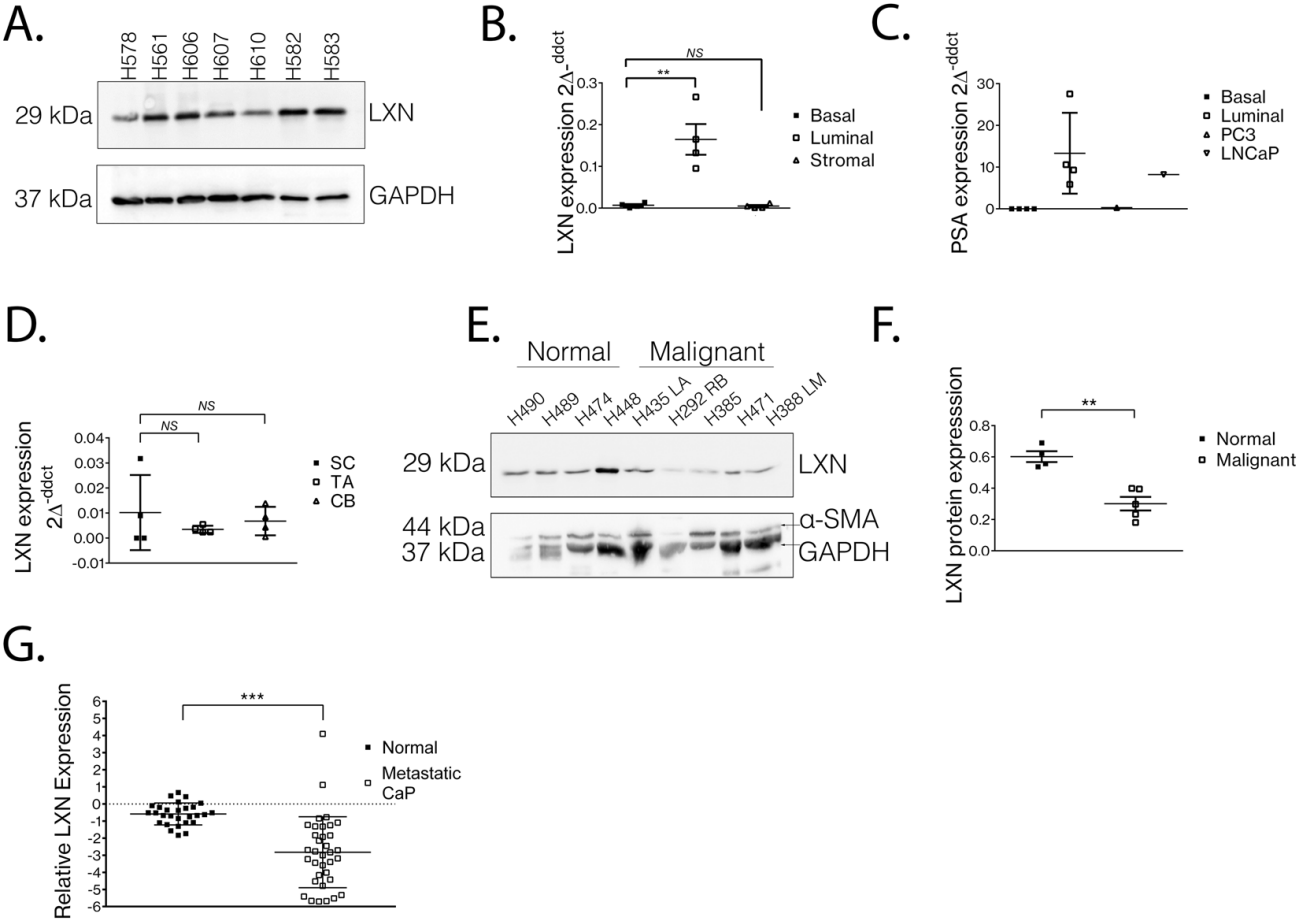
LXN is highly expressed in non-malignant prostate luminal cells and repressed in high Gleason grade tumours. (**A**) Western blot data to assess LXN protein expression in the non-malignant human prostate. Data from seven different tissue specimens GAPDH serves as a loading control*. (**B**) Direct cell sorting from uncultured human prostate tissue into basal, luminal and stromal fractions reveals that LXN is predominantly expressed in prostate luminal cells and 24.25 ± 5.41-fold greater than basal cells (*p* < *0*.*01*). RT-PCR analysis from four separate tissue samples. (**C**) mRNA expression analysis of the luminal specific marker PSA confirms the technical success of the FACS approach. The PC3 and LNCaP cell lines were used as basal and luminal cell controls, respectively. (**D**) Comparison of LXN gene expression following sub-fractionation of primary cultures of basal epithelial cells into stem cells (SC), transit amplifying (TA) and committed basal (CB) cells. Data is representative of four different primary culture samples. (**E**) Western blot to compare LXN protein expression in normal and malignant prostate tissue. α-SMA and GAPDH were used as indicators of relative stromal abundance and total protein loading respectively*. (**F**) Scanning densitometry of (**E**). (**G**) Gene expression analysis Grasso *et al*. prostate database reveals that LXN mRNA expression is significantly reduced in metastatic prostate disease compared to normal prostate (*p* < *0*.*01*). mRNA expression analysis is presented as 2 Δ^ddct^ (LXN/RPLP0). *Full length, uncropped blots from (**A,F**) can be viewed in Supplementary Fig. S6.

To determine whether LXN is expressed in the human prostate we first screened several anti-LXN antibodies and were able to validate a suitable candidate for detection of LXN protein expression via Western blotting (Supplementary Fig. S1A–E). Protein expression analysis of seven individual non-malignant prostate tissue homogenates revealed that LXN is expressed in the normal prostate (Fig. 1A). Prostate tissue is comprised of basal epithelial and terminally differentiated luminal cells surrounded by a fibromuscular stromal layer^19,20^. In the absence of a suitable antibody for immunohistochemistry (IHC), we isolated cells directly from uncultured human prostate tissue and purified them in to separate basal, luminal and stromal cell fractions using fluorescence-activated cell sorting (FACS) (Supplementary Fig. S1F). Analysis of LXN mRNA expression in these subsamples revealed that whilst LXN is expressed in all fractions, luminal cells express 24.25 ± 5.41-fold (*p* = *0*.*004)* greater levels than basal cells. Stromal cells also express LXN, but at similar levels to basal cells (Fig. 1B). Prostate specific antigen (PSA) and androgen receptor (AR) are luminal cell markers and confirmed the stringency of our cell sorting strategy when compared to control cell lines: PC3 cells (basal-type) and LNCaP (luminal-type) cells (Fig. 1C and Supplementary Fig. S1G). Oldridge *et al*. reported LXN mRNA expression to be significantly lower in normal prostate tissue stem cells compared to more differentiated basal cells^18^. However, when we analysed the mRNA expression levels of LXN across the basal epithelial cell hierarchy (stem cell, transit amplifying and committed basal^19^), we found that prostate tissue stem cells do not express significantly different levels of LXN compared to more differentiated basal cells (Fig. 1D). LXN expression has been reported to be repressed in many solid tumours including thyroid, hepatic and pancreatic carcinomas^13,15,21^. To understand whether the same expression patterns were observed in normal versus malignant prostate, we homogenised several normal and several high Gleason (>G8) grade prostate tissue specimens and compared the protein expression of LXN via Western blotting (Fig. 1E). To control for the relative abundance of stroma within each sample, we probed for smooth muscle actin (SMA) in addition to GAPDH. Our data suggest that LXN expression is repressed in cancerous prostate compared to normal (50%, p = *0*.*001*, Fig. 1E,F). In-keeping with these findings we found samples > GL7 from the Taylor *et al*.^22^ database also showed significant reduction in LXN mRNA expression compared to normal or lower Gleason grade tumours (Supplementary Fig. S1H)^22^. In order to understand the wider relevance of this finding, we interrogated a publicly available dataset (Grasso *et al*. 2012) and found LXN mRNA levels to be significantly downregulated in metastatic prostate cancer specimens when compared to normal prostate tissue (Fig. 1G)^23,24^.

## LXN is secreted by prostate epithelial cells

**Figure 2:**
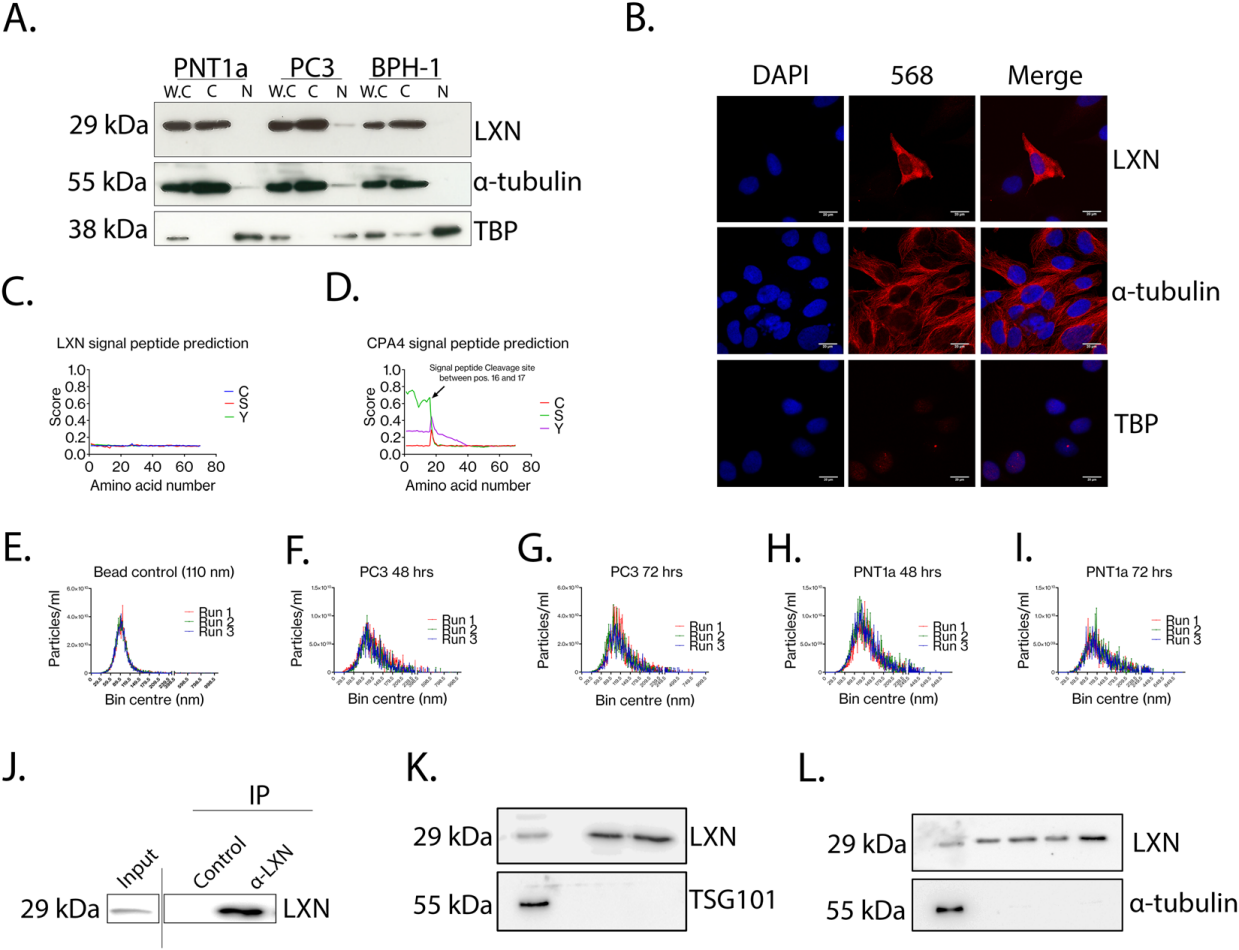
LXN is cytosolic and also secreted. (**A**) Western blot analysis from subcellular fractionation studies in three prostate epithelial cell lines to determine LXN subcellular localisation. Representative blot from four independent experiments/cell line. α-tubulin and TBP indicate the purity of the cytosolic and nuclear fractions respectively. W.C (whole cell lysate), C (cytosolic), N (nuclear). See Supplementary Fig. S7 for uncropped original blots at 3 different exposures. (**B**) Confocal microscopy depicting Immunocytochemistry of PNT1A cells following transient overexpression of LXN-HA (upper panel). α-tubulin (middle panel) and TBP (lower panel) demonstrate the unbiased accessibility of both the nuclear and cytosolic compartments using this approach (Scale bar = 20 µM). (**C**,**D**) *in silico* analysis using SignalP 4.0 software to predict the occurrence of signal peptides in protein amino acid sequences reveals that LXN does not contain a predicted Signal peptide, whereas CPA4 does. (**E**) Nanoparticle tracking analysis (NanoSight NS300, Malvern) of positive control beads consistent with the size of exosomal particles (110 nM diameter) to determine that EVs purified were consistent with the size of exosomes in (**F**,**G**,**H** and **I**) which represent the purified EV fractions from PC3 and PNT1A cells respectively. (**J**) Enrichment of LXN from cell culture conditioned media following immunoprecipitiation using a LXN specific antibody compared to an isotype control and conditioned media control (input). This gel has been cropped to bring different parts of the same gel together for concisness and clarity. See Supplementary Fig. S2 for the full blot with all additonal controls. (**K**) Western blots demonstrating the relative abundance of LXN within purified EVs versus soluble secreted fractions (EV depleted media). TSG101 serves as a marker for successful isolation of endo/lysosomal material from the extracellular space. (**L**) Western blot of EV depleted media at two separate time points in PNT1A or PC3 cell lines. Tubulin was used to demonstrate the absence of any contaminating cellular material. Full length uncropped blots for (**K,L**) can be viewed in Supplementary Fig. S8A,B.

LXN has been reported to be a predominantly cytosolic protein in the leukeamic cell line FDC-P1, in murine HSCs and also in primary human breast adenocarcinoma^14,17,25^. However, Oldridge *et al*. (2013) presented preliminary data to suggest that LXN may be a nuclear protein in the prostate. To test the findings of Oldridge *et al*. we characterised the subcellular distribution of LXN in human prostate cells. Interestingly, after purification of both nuclear and cytosolic fractions from several prostate epithelial cell lines we found LXN to be exclusively cytosolic (Fig. 2A) even after overexposure of Western blots (Supplementary Fig. S2A). Any LXN positivity in the nuclear fraction was accompanied by α-tubulin positivity, suggesting a small amount of cytosolic contamination (Supplementary Fig. S2A). These findings are consistent with many other LXN studies, but oppose the results of Oldridge *et al*. As the LXN antibody was unsuitable for use in immunocytochemistry, we transiently overexpressed LXN with a haemagglutinin tag (LXN-HA) to observe LXN subcellular distribution using a highly specific anti-HA antibody (Fig. 2B and Supplementary Fig. S2B). We utilised α-tubulin, a cytoskeletal protein and TATA binding protein (TBP), a general transcription factor to demonstrate unbiased antibody access to the cytosolic and nuclear subcellular compartments following fixation and permeabilisation of cells. Using this approach, LXN-HA appeared to be predominantly cytosolic, with a diffuse cytosolic expression pattern. There was a lack of convincing plasma membrane staining, and no puncta typical of endosome location or staining of reticular networks consistent with ER, Golgi or mitochondrial localisation. To take an unbiased approach in the characterisation of LXN subcellular distribution, we also tested to see whether LXN is a secreted protein. We were able to immunoprecipitate LXN from prostate epithelial cell culture conditioned medium using the anti-LXN antibody in comparison to an isotype matched antibody control (Fig. 2J and Supplementary Fig. S2C). *In silico* analysis of the LXN amino acid sequence predicted that LXN does not contain a signal peptide (Fig. 2C,D)^26^. From these analyses we hypothesised that LXN might be secreted non-classically via extracellular vesicles (EV). To test this hypothesis, we purified EVs from cell-free PC3-conditioned medium and demonstrated the presence of predominantly small nano-particles in these isolates using Nanoparticle tracking analysis (Fig. 2E–L and Supplementary Fig. 2D,E). We tested LXN expression within the EV isolates and the soluble secreted fraction (Fig. 2K,L) and found that whilst we could detect a small amount of LXN in the total purified EV fraction, LXN was mostly present within the soluble secreted fraction (Fig. 2K). To exclude the possibility of LXN passively diffusing from dead or dying cells, we used tubulin to confirm that no contaminating intracellular proteins were present in the EV isolate or secreted fraction (Fig. 2L).

## LXN reduces the plating efficiency of prostate luminal cells

**Figure 3:**
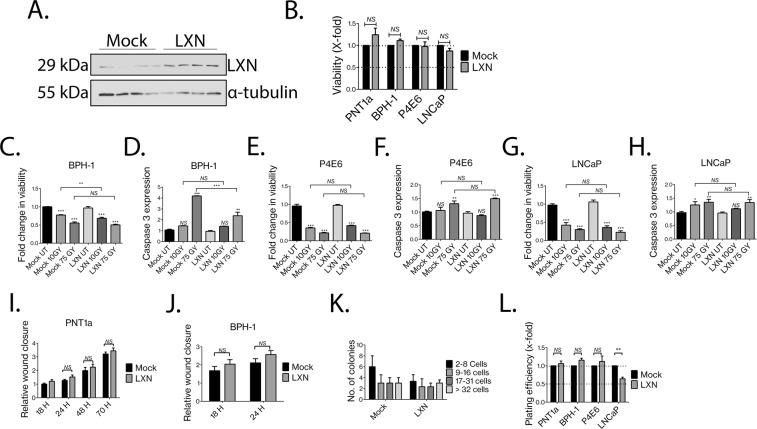
LXN reduces the plating efficiency of prostate luminal cells. (**A**) Representative image from a Western blot (n = 4) showing robust stable overexpression of LXN protein in prostate epithelial cell lines following lentiviral transduction. See Supplementary Fig. S9 for full length uncropped blot images (**B**). Cell viability assays demonstrating the effects of LXN overexpression in non-malignant (PNT1A and BPH-1) and malignant (P4E6 and LNCaP) prostate epithelial cell lines. (**C–H**) The effects of low (10 Gy) and high (75 Gy) doses of γ-irradiation on the viability (**C**,**E**,**G**) and apoptosis (**D**,**F**,**H**) of prostate epithelial cell lines. (**I**,**J**) Wound scrape assays to determine the effects of LXN overexpression on wound healing and motility in BPH-1 and PNT1A cell lines. (**K**) Effect of LXN overexpression on the colony forming effeciency of primary prostate epithelial data representative of 3 patient samples (**L**). Plating efficiency assays in prostate epithelial cell lines (*n* = *3*). *NS* = *non-significant*. *p* = *0*.*0013*.

LXN expression reportedly affects the growth of several different tumours. For instance, overexpression of LXN decreases proliferation in Lymphoma^11^. Furthermore, LXN can play a tumour suppressive role in several different adenocarcinomas^13,15,18,21^. To determine the biological effects of LXN in prostate epithelial cells, we generated lentiviral vectors to induce robust and stable overexpression of LXN in normal (PNT1A), Benign (BPH-1) and cancer cell lines P4E6 (basal-like) and LNCaP (luminal –like) (Fig. 3A). Compared to cells transduced with lentiviral control particles (Mock), overexpression of LXN did not affect the viability of any of the cell lines tested, including the luminal-like cancer cell line, LNCaP (Fig. 3B). LXN also had no significant effects on the proliferation of cell lines (Supplementary Fig. 3A). You *et al*.^17^ reported that LXN sensitises leukeamic cells to the effects of DNA damage following ionising radiation^17^. To understand if the same effects were seen when LXN was overexpressed in human prostate cells, we measured viability and apoptosis following exposure to a low (10 Gy) or high (75 Gy) dose of γ-irradiation. (Fig. 3C–H). Confirmation of the induction of DNA damage response pathways were assessed by γ-H2AX foci (data not shown). We observed a dose-dependent decrease in cell viability following DNA damage in BPH-1, P4E6 and LNCaP cells (Fig. 3C,E,G), which correlated with a dose-dependent increase in apoptosis (Fig. 3D,F,H). However, overexpression of LXN had no significant effect on these processes. We did however see a small decrease in the viability of LXN overexpressing BPH-1 cells, compared to mock transduced controls, but this did not correlate with a corresponding increase in apoptosis. Additionally, we saw a decrease in apoptosis in LXN overexpressing BPH-1 cells, treated with 75 Gy of radiation, yet viability was not significantly altered. Oldridge *et al*. showed that transient knockdown of LXN in the PNT1A prostate epithelial cell line repressed motility by 62.5%, and that subsequent transient overexpression of HA-tagged LXN increased motility by 29%^18^. Motility changes in stably transfected BPH-1 and PNT1A cells overexpressing LXN indicated a trend towards increased motility. However, we were unable to obtain statistical significance in any of the cell lines tested (Fig. 3I,J and Supplementary Fig. 3B). When the effects of LXN overexpression on the colony forming efficiency of primary prostate epithelial cells (which are predominately basal) were tested, it was found that LXN overexpression had no significant effect on either colony number or size (Fig. 3K). Interestingly, whilst LXN had little effect on basal cells of non-malignant or malignant origin, the plating efficiency of the luminal-like cancer cell line, LNCaP, which expressed undetectable levels of endogenous LXN, was significantly reduced (35.5% ± 4.1%, *p* = *0*.*001*3*)* in the LXN stable overexpression line compared to mock transduced cells (Fig. 3L).

## LXN is directly upregulated by atRA in prostate cells

**Figure 4:**
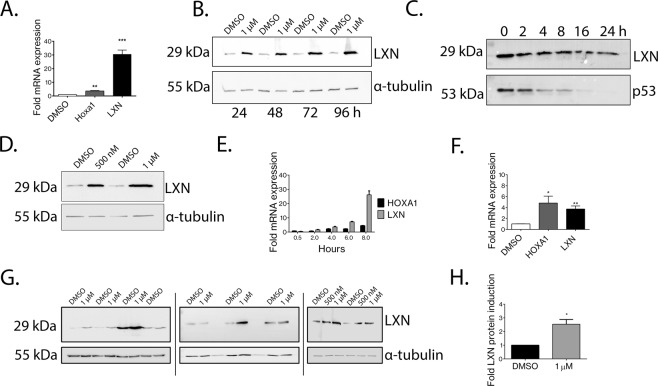
LXN is directly upregulated by the differentiation agent atRA in human prostate epithelial cells. (**A**) Significant induction of LXN (30.57 ± 3.011-fold *p* = *0*.*0006)* and HOXA1 (3.65 ± 0.3421-fold *p* = *0*.*0015)* mRNA expression in PNT1A cells 8 hrs after treatment with 1 µM atRA. RT-PCR analysis from 3 independent experiments. (**B**) A time-course showing significant induction of LXN protein expression from 24 hrs after treatment with 1 µM atRA. See Supplementary Fig. S10A,B for uncropped blots. (**C**) Protein half-life assay to determine the stability of LXN over a 24 hr time-course following treatment of PNT1A cells with 10 µM of the protein synthesis inhibitor anisomycin. The relatively unstable protein p53 serves as a positive control. See Supplementary Fig. S10C,D for uncropped blot images. (**D**) Dose dependent induction of LXN protein expression following treatment with 500 nM or 1 µM atRA. See Supplementary Fig. S10D,E for uncropped blot images. (**E**) Induction of LXN and HOXA1 mRNA expression over an 8 hr time-course following treatment with 1 µM atRA in PNT1A cells (n = 3). (**F**) Significant induction of HOXA1 (4.83-fold ± 1.26-fold *p* = *0*.*0158*) and LXN (3.73 ± 0.57-fold *p* = *0*.*0015)* mRNA expression in primary human prostate epithelial cells 24 hrs after treatment with 500 nM atRA (n = 9). (**G**) Assessment of LXN protein expression 72 hrs after treatment with 500 nM atRA in 9 individual primary prostate epithelial cell samples. Data obtained from 3 separate blots (indicated by hatched lines) (See Supplementary Fig. S11 for full sized uncropped blots and multiple exposures). Scanning densitometry of (**F**) revealed LXN to be significantly induced 2.54 ± 0.34- fold *p* = *0*.*0313* (n = 9).

LXN expression has been reported to be responsive to retinoic acid (atRA) treatment, for example, by proteomic analysis of atRA treated MCF-7 (breast epithelial) cells^27^. Moreover, LXN mRNA was reported to be upregulated in response to retinoic acid in prostate epithelial cell lines^18^. Considering the findings from previous studies, we decided to characterise the LXN response to atRA more thoroughly within the human prostate. To investigate the dynamics of the LXN response to atRA in the non-malignant prostate we employed the PNT1A cell line. We observed robust and significant induction of LXN mRNA expression within eight hours of treatment with 1 µM atRA (30.57 ± 3.01-fold, *p* = *0*.*0006)*. In comparison, a positive control gene (HOXA1), was only upregulated (3.65 ± 0.34-fold, *p* = *0*.*0015)* at this time-point (Fig. 4A). Furthermore, treatment of PNT1A cells with 1 µM atRA resulted in induction of LXN protein expression after 24 hours, which continued to accumulate to 96 hours after treatment (Fig. 4B). These data are in accordance with the long-half-life of LXN (>24 hours) as measured in anisomycin half-life assays (Fig. 4C). Additionally, treatment of PNT1A cells for 24 hours with either 500 nM or 1 µM atRA resulted in strong induction of LXN protein expression in a dose dependent manner (Fig. 4D). We then attempted to compare the induction of LXN mRNA expression with that of HOXA1 which is directly upregulated by atRA^28–30^. We found that LXN and HOXA1 were both induced from two hours following atRA treatment. The levels of LXN mRNA expression accumulated over an eight-hour time-course (Fig. 4E), peaking at 24 hours whilst HOXA1 expression peaked between eight & 24-hours (Supplementary Fig. S4A). We next validated our findings in near-patient, non-malignant, primary epithelium. Here, both HOXA1 and LXN mRNA were significantly upregulated following atRA treatment (4.83 ± 1.26-fold, *p* = *0*.*0158* and 3.73 ± 0.57-fold, *p* = *0*.*0015* respectively, n = 9), and in six out of seven primary samples, LXN protein expression was increased after 72 hours treatment with 1 µM retinoic acid (average 2.54 ± 0.34-fold induction, *p* = *0*.*031)* (Fig. 4F,G,H respectively).

## LXN indirectly induces a pro-inflammatory transcriptome signature

**Figure 5:**
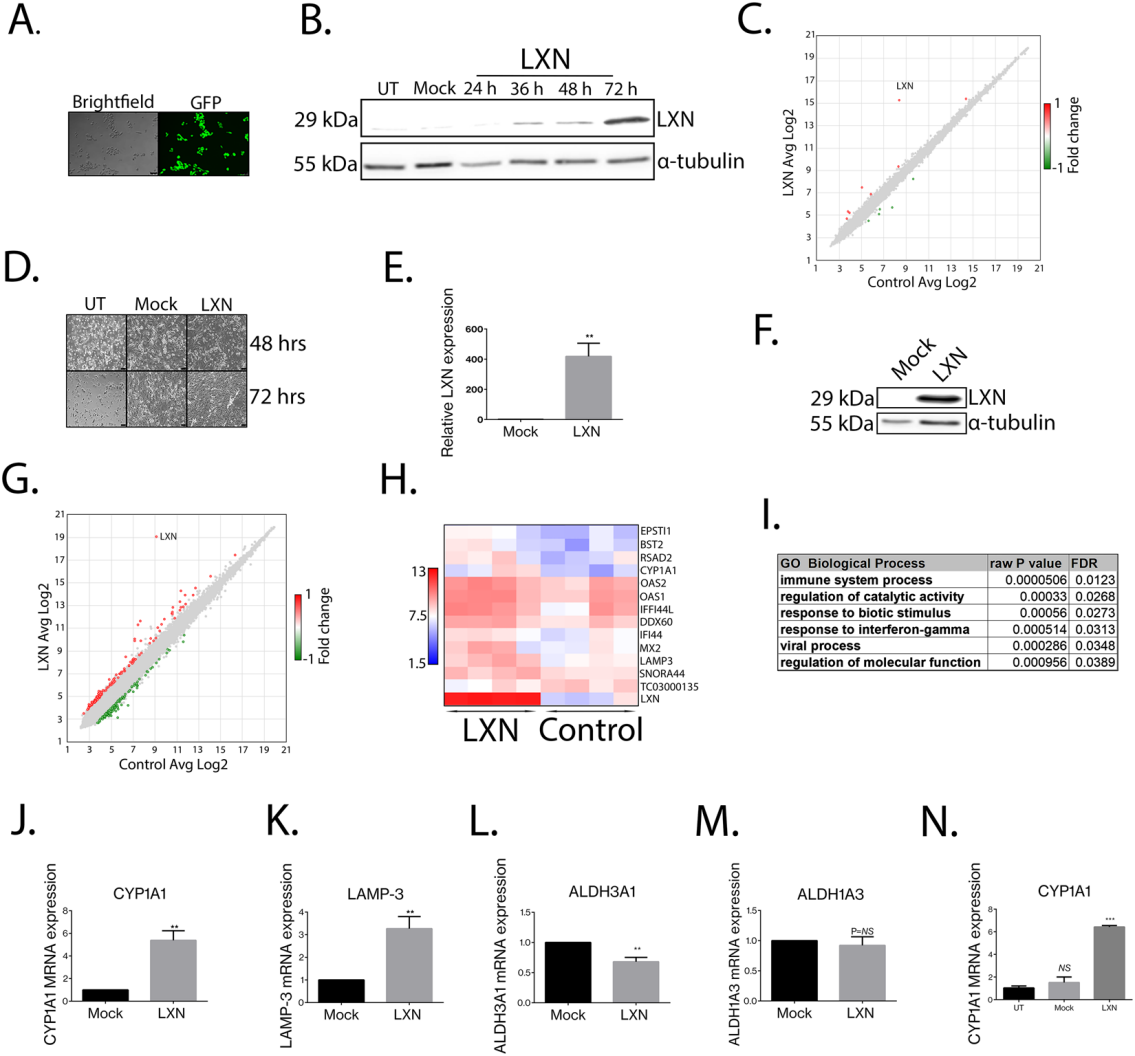
LXN indirectly induces a pro-inflammatory trancriptome signature. Lentiviral particles containing Mock control or LXN both with a GFP reporter gene were used to track overexpression of proteins after viral transduction. (**A**) Representative image of GFP expression in live prostate cells 36 hrs after viral transduction using an epifluorescence microscope. (**B**) Western blot data depicting a time-course to determine the earliest time after lentiviral transduction at which LXN is overexpressed. α-tubulin serves as a loading control. (**C**) Representative scatter plot to show differentially expressed genes 36 hrs after transduction of LXN (cut off was ± 2-fold change *p* < *0*.*05)*. LXN was found to be overexpressed 118.31-fold, *p* = *0*.*0075*, *(n* = *4)* at this time-point after viral transduction. (**D**) Bright-field images depicting primary prostate cells 48 & 72 hrs after selection in puromycin. Cells were transduced with control particles (Mock) or LXN lentiviral particles both containing a puromycin resistance cassette. Non-transduced primary prostate epithelia (UT) demonstrate the success of viral transduction. (**E**) Lentiviral transduced cells were confirmed on average to be overexpressing LXN using RT-PCR (418 ± 87.92-fold, *p* = *0*.*0032*, n = 4). (**F**) Western blot demonstrating that lentiviral transduced primary prostate epithelia overexpress LXN protein compared to cells transduced with control particles (α-tubulin serves as a loading control). (**G**) Representative scatter plot to show differentially expressed genes 1 week after transduction of LXN (cut off was ± 2-fold change, *p* < *0*.*05*). (**H**) Representative heatmap to demonstrate the largest gene expression changes in individual samples. (**I**) Funtional annotation of differentially expressed genes in response to LXN overexpresion (see Supplementary Table 1 for detailed list). (**J**,**K**,**L**) Independent validation of the effects of LXN overexpression on CYP1A1, LAMP-3 and ALDH3A1 in non-malignant primary prostate epithelia (n = 9) (**M**). ALDH1A3 mRNA expression was not found to be significantly affected by LXN overexpression. (**N**) Stable overexpression of LXN in the luminal-like cancer cell line LNCaP resulted in significant induction of CYP1A1 mRNA expression compared to untransduced cells (UT) or cells transduced with control particles (6.42 ± 0.03 -fold, *p* < *0*.*000*, n = 3). mRNA expression is presented as fold change relative to cells transduced with control particles (Mock).

To further explore the function and mechanism of action of LXN in the human prostate we chose a transcriptomic approach. To examine potentially direct effects of LXN on total cellular gene expression, we overexpressed LXN, along with a GFP reporter gene, in non-malignant primary prostate epithelial cells by lentiviral transduction of the heterogeneous cell population. We utilised GFP positivity and subsequent Western blotting for LXN, to confirm the earliest time at which LXN is significantly overexpressed at the protein level. This was found to be 36 hours post transduction (Fig. 5A,B and Supplementary Fig. 5A). Subsequent transcriptome analyses of these samples revealed that short-term overexpression of LXN did not significantly alter total cellular gene expression. This is further evidence in support of the predominantly cytosolic and extracellular localisation of LXN and lack of any predicted nuclear localisation signal (Fig. 2). In accordance with our PCR and Western blot data, LXN was observed to be upregulated 118.31-fold, *p* = *0*.*0075* (n = 4) 36 hours after lentiviral transduction, compared to cells transduced with control particles in the transcriptome arrays (Fig. 5C and Supplementary Fig. 5C–E).

To explore the long-term, and therefore indirect effects of LXN on gene expression, we selected non-malignant primary prostate epithelial cells overexpressing LXN or mock control lentiviral particles using a puromycin resistance cassette (Fig. 5D). Cells were confirmed to be overexpressing LXN via RT-PCR (average increase of 418 ± 87.92-fold, *p* = *0*.*0032*, n = 4) and Western blotting (Fig. 5E,F respectively). To identify genes that are modulated by stable overexpression of LXN, we again performed transcriptome analysis. Of 13570 genes, we identified 256 (0.19%) that passed the criteria for selection (<−2 or >2 fold, p < 0.05). Of those, 131 (51.17%) were found to be up-regulated and 125 (48.83%) to be down-regulated in response to stable LXN overexpression (Fig. 5G and Supplemental Fig. S5C–E). Functional annotation of the differentially expressed genes included those involved in catalytic processes, immune cell differentiation and inflammation, such as the IFN-γ response (Fig. 5H,I and Supplementary Table S2). To assess the validity of these findings we utilised the Cancer genome atlas (TCGA) database to assess concurrence of LXN alterations in human cancer gene databases with the most differentially expressed genes in our transcriptome arrays^31,32^. 23 of 86 genes showed a statistically significant relationship (Table 1). Additionally, we decided to empirically validate a number of genes by using additional non-malignant primary prostate epithelial samples. We focused on differentially expressed genes linked to retinoic acid signalling and metabolism as our data indicate a role for LXN in the response to retinoic acid. We found that LXN over expression caused significant upregulation of CYP1A1 (5.8 ± 0.85 -fold and LAMP-3 3.27 ± 0.53-fold) and repression of ALDH3A1 by 44% ± 4.1% (Fig. 5J, K,L respectively). However, we were unable to reproduce a significant effect of LXN overexpression on ALDH1A3 expression after repeated measurement (0.92 ± 0.14-fold, *p* = *NS*, n = 9 and Fig. 5M). Finally, long-term overexpression of LXN in the luminal CaP cell line LNCaP also resulted in significant upregulation of CYP1A1 when compared to untransduced cells or cells transduced with control particles (6.42 ± 0.03-fold, *p* < *0*.*0001*, n = 3) (Fig. 5N).

**Table 1:** TCGA data demonstrating the proportion of genes found to be indirectly regulated by LXN overexpression in prostate epithelial that also show concurrent genetic alterations with LXN in human cancer^31,32^.

Differentially expressed genes	log odds ratio	p-value	adjusted p- value	Tendency
LAMP3	>3	<0.001	<0.001	CO-OCCURRENCE
PLSCR1	>3	<0.001	<0.001	CO-OCCURRENCE
PARP14	>3	<0.001	<0.001	CO-OCCURRENCE
ZPLD1	>3	<0.001	<0.001	CO-OCCURRENCE
LINC02085	>3	<0.001	<0.001	CO-OCCURRENCE
DDX60L	>3	<0.001	<0.001	CO-OCCURRENCE
DDX60	>3	<0.001	<0.001	CO-OCCURRENCE
SNORD72	>3	<0.001	<0.001	CO-OCCURRENCE
43348	>3	<0.001	<0.001	CO-OCCURRENCE
PHGDH	>3	<0.001	<0.001	CO-OCCURRENCE
FAM129A	>3	<0.001	<0.001	CO-OCCURRENCE
BST2	>3	<0.001	<0.001	CO-OCCURRENCE
HIST2H4B	>3	<0.001	<0.001	CO-OCCURRENCE
HIST2H4A	>3	<0.001	<0.001	CO-OCCURRENCE
STAT1	>3	<0.001	<0.001	CO-OCCURRENCE
MAGE4D	>3	<0.001	<0.001	CO-OCCURRENCE
SNORA11D	>3	<0.001	<0.001	CO-OCCURRENCE
SNORA11E	>3	<0.001	<0.001	CO-OCCURRENCE
SULT1E1	>3	<0.001	<0.001	CO-OCCURRENCE
MAGED4B	>3	<0.001	<0.001	CO-OCCURRENCE
RSAD2	>3	<0.001	0.001	CO-OCCURRENCE
HERC5	>3	<0.001	0.003	CO-OCCURRENCE
TAPBPL	>3	<0.001	0.003	CO-OCCURRENCE
GPNMB	2.785	<0.001	0.003	CO-OCCURRENCE
GP1BB	>3	<0.001	0.007	CO-OCCURRENCE
KMO	2.563	<0.001	0.011	CO-OCCURRENCE
ASNS	3	<0.001	0.028	CO-OCCURRENCE
LBH	>3	<0.001	0.157	CO-OCCURRENCE
MX1	>3	<0.001	0.211	CO-OCCURRENCE
IFIH1	2.43	<0.001	0.507	CO-OCCURRENCE
CYP1A1	2.695	<0.001	0.956	CO-OCCURRENCE
TNRSF10D	2.272	<0.001	0.999	CO-OCCURRENCE
MIR421	>3	<0.001	1	CO-OCCURRENCE
RRBP1	2.582	<0.001	1	CO-OCCURRENCE
SNORD114-6	2.981	<0.001	1	CO-OCCURRENCE
IFI44L	2.33	<0.001	1	CO-OCCURRENCE
OASL	2.847	0.001	1	CO-OCCURRENCE
GBP4	2.175	0.002	1	CO-OCCURRENCE
COL26A1	2.084	0.002	1	CO-OCCURRENCE
PIP5K1B	2.441	0.003	1	CO-OCCURRENCE
MX2	2.4	0.003	1	CO-OCCURRENCE
BIRC3	1.443	0.004	1	CO-OCCURRENCE
H1F10	>3	0.005	1	CO-OCCURRENCE
IFI44	2.251	0.007	1	CO-OCCURRENCE
IRF1	2.904	0.011	1	CO-OCCURRENCE
ATF3	2.063	0.018	1	CO-OCCURRENCE
MIR503	2.642	0.02	1	CO-OCCURRENCE
EPSTI1	2.373	0.024	1	CO-OCCURRENCE
SLC6A9	2.316	0.033	1	CO-OCCURRENCE
PARP6	2.21	0.038	1	CO-OCCURRENCE
OAS3	2.028	0.124	1	CO-OCCURRENCE
OAS2	1.948	0.171	1	CO-OCCURRENCE
MIR548F4	2.108	0.198	1	CO-OCCURRENCE
TAGLN	1.733	0.225	1	CO-OCCURRENCE
KIF20A	1.561	0.847	1	CO-OCCURRENCE
OAS1	1.414	0.885	1	MUTUAL EXCLUSIVITY
CHAC1	>3	0.895	1	MUTUAL EXCLUSIVITY
RCAN3	>3	0.936	1	MUTUAL EXCLUSIVITY
CCL5	>3	0.936	1	MUTUAL EXCLUSIVITY
SNORA44	>3	0.936	1	MUTUAL EXCLUSIVITY
SNORA61	>3	0.936	1	MUTUAL EXCLUSIVITY
SNORA16A	>3	1	1	MUTUAL EXCLUSIVITY
SNHG12	>3	1	1	CO-OCCURRENCE
RNA5S2	>3	1	1	CO-OCCURRENCE
RNA5S3	>3	1	1	CO-OCCURRENCE
RNA5S1	>3	1	1	CO-OCCURRENCE
RNA5S4	>3	1	1	CO-OCCURRENCE
RNA5S5	>3	1	1	CO-OCCURRENCE
RNA5S11	>3	1	1	CO-OCCURRENCE
RNA5S12	>3	1	1	CO-OCCURRENCE
RNA5S13	>3	1	1	CO-OCCURRENCE
RNA5S14	>3	1	1	CO-OCCURRENCE
RNA5S15	>3	1	1	CO-OCCURRENCE
RNA5S16	>3	1	1	CO-OCCURRENCE
RNA5S6	>3	1	1	CO-OCCURRENCE
RNA5S7	>3	1	1	CO-OCCURRENCE
RNA5S8	>3	1	1	CO-OCCURRENCE
RNA5S10	>3	1	1	CO-OCCURRENCE
RNA5S17	>3	1	1	CO-OCCURRENCE
RNU6-1176P	>3	1	1	CO-OCCURRENCE
RNU6-1291P	>3	1	1	CO-OCCURRENCE
RNU61098P	>3	1	1	CO-OCCURRENCE
MIR7976	>3	1	1	CO-OCCURRENCE
MT-TD	>3	1	1	CO-OCCURRENCE

## Discussion

LXN has been associated with numerous very different malignancies. For example, LXN expression is downregulated in lymphoma, gastric carcinoma and thyroid carcinoma^11,21,33^ and loss of LXN expression correlates with diverse biological effects in different cancer cell lines^15,17^. Such findings suggest that LXN may be a multi-functional protein or exhibit cell type specific effects. Despite multiple attempts to determine a role for loss of LXN expression in adenocarcinoma, thorough characterisation of LXN in normal epithelia or malignancy is still lacking. To further our understanding of LXN function in the normal and malignant prostate, we set out to understand where within the prostate cellular hierarchy LXN is expressed, investigate its subcellular localisation and characterise its biological role. We describe here for the first time, using an antibody extensively validated for use in Western blotting, that LXN is indeed robustly expressed in the human prostate. Furthermore, we found that, whilst LXN is expressed throughout the prostate, it is most highly expressed in luminal cells, suggesting a role in luminal cell function and/or differentiation. When we compared the expression levels of LXN between non-malignant and malignant prostate tissue, we found that LXN was significantly repressed in high Gleason grade cancers. Additionally, LXN expression was undetectable in two luminal type CaP lines (LNCaP and VCaP). These findings are interesting given that histologically, prostate cancers are predominantly luminal in phenotype, where LXN would appear to be most highly expressed, based on its normal epithelial cell expression^19^. Therefore, it is possible that transformed luminal cells may acquire a survival benefit by reducing levels of LXN. Finally, we found that metastatic prostate cancers also showed a striking down-regulation of LXN compared to non-malignant specimens. These data further raise the possibility that LXN expression is lost as CaP progresses. With further progression CaP cells become less differentiated and the luminal phenotype is lost, providing one explanation for loss of LXN expression.

Endogenously expressed LXN was observed to be cytosolic in prostate epithelia after subcellular fractionation, in keeping with the lack of any predicted NLS. These observations are in accordance with predictions from other studies^15,17,33^, but are in disagreement with the findings of Oldridge *et al*. whose preliminary findings suggest that ectopically expressed LXN-HA is a predominantly nuclear protein in prostate cells^18^. Our immunocytochemistry experiments also utilised ectopic overexpression of LXN-HA which is known to cause protein mislocalisation^34–36^. Despite this we still observed LXN to be predominantly cytosolic. Additionally, we now provide the first empirical evidence to suggest that LXN is secreted since we could find high levels in the conditioned media of several prostate epithelial cell lines. Given that our *in silico* analysis predicted that LXN lacks a signal peptide, we hypothesised that LXN might enter the extracellular space via EV release. Despite obtaining highly pure EV isolates from PNT1A or PC3 cell lines, we only found a small fraction of the total amount of secreted LXN to be present within vesicle isolates, and that there was negligible depletion of LXN from the secretome following ultracentrifugation at 100,000 x g. Therefore, it is more likely that LXN is secreted from cells via another mechanism, and future work would seek to determine the mechanism of LXN secretion. It is interesting to note that so-called leaderless proteins (lacking a signal peptide) can be secreted via numerous non-exosomal pathways^37^.

Next, we sought to determine the biological effects of LXN in prostate epithelial cells. Interestingly, robust and stable overexpression of untagged LXN did not significantly affect cell proliferation, viability or motility of prostate cell lines. LXN also did not appear to influence sensitivity to ionising radiation which is in contrast to the findings of You *et al*.^17^ who reported that LXN sensitises leukeamic cells to the effects of DNA damage following ionising radiation^17^. For these experiments we used two doses of ionizing radiation. The first, 10 Gy has been used to induce DNA damage in prostate epithelial cell lines^38,39^. However, whilst there was a clear trend, we were unable to significantly reduce cell viability or increase apoptosis at this dose. In this setting LXN induced increases in sensitivity to ionizing radiation would likely be detectable compared to time-matched negative controls. The second dose 75 Gy is higher than usually performed in cell culture experiments and reflects the total dose of radiation that patients undergoing radiation therapy receive over the course of several treatments^40^. Therefore, whilst our data suggest that LXN does not increase sensitivity to ionizing radiation at the doses used we acknowledge that our use of 75 Gy is quite high and that it is possible that LXN driven changes in the sensitivity to ionizing radiation may occur at lower doses. Intriguingly, LXN overexpression in primary prostate epithelial cells (which have a basal phenotype) did not significantly alter colony forming ability (an indicator of ‘stemness’) but did significantly reduce the plating efficiency of the luminal-type LNCaP cell line. Therefore, whilst LXN overexpression did not alter the viability of LNCaP cells, overexpression inhibited the ability of a subset of cells to adhere and therefore proliferate in culture. Such findings suggest that LXN may act as a tumour suppressor by reducing the ability of cancer cells to colonize metastatic sites. These data, together with our finding that LXN is mostly expressed in luminal cells and is secreted, suggest that LXN is likely to function predominantly within the prostate lumen or within luminal cells. Moreover, given that LXN appears to be downregulated in CaP, our data indicates that loss of LXN in luminal cells may be associated with malignant progression.

There is increasing evidence to suggest that LXN may be retinoid-responsive^18,27^. We have demonstrated for the first time that LXN is induced by retinoic acid (atRA) at both the mRNA and protein level, in both human prostate cell lines and primary prostate epithelia. LXN is directly and rapidly upregulated by atRA with similar kinetics to those of HOXA1, a well characterised primary gene expression response to atRA^30^. Interestingly, we found LXN protein to be extremely stable with a half-life exceeding 24 hours (a proteomic study in a bladder cancer cell line suggested 48 hour half-life^28,41^). These findings suggest that LXN may play a direct and lasting downstream role in the biological response to retinoids. One possible mechanism by which LXN may influence or regulate retinoic acid metabolism is through its proposed enzyme inhibitory function^42^. Given that LXN is induced directly by retinoic acid, it may form part of a feedback loop and function to inhibit enzymes involved in the metabolism of pre-cursor retinoids into atRA, thus helping to regulate atRA responses.

Transcriptome analyses of short-term overexpression of LXN did not yield any significant changes in gene expression. This finding, along with our demonstration that LXN is a secreted protein with limited access to the nuclear compartment, indicate that LXN does not function as a direct regulator of gene expression within the prostate epithelium. In contrast, long-term overexpression of LXN did result in transcriptomic changes, revealing that LXN regulates gene expression indirectly. Functional annotation of genes indirectly regulated by LXN overexpression include those involved in catalytic processes, and genes involved in immune cell function and inflammatory processes such as the response to IFN. These findings are supported by several studies linking LXN expression to inflammatory responses^42–46^. For example siRNA knockdown of MAFB in mouse macrophages resulted in significant anti-inflammatory responses which included significant reductions in levels of LXN, OAS1 OAS2, MX2 and IFNl44^44^. In another study, treatment of retinal pigment epithelium (RPE) cells with amyloid-*β*_1-40_, a known inflammatory trigger in Age-related macular degeneration (AMD) caused a significant induction of inflammatory genes such as BST2, STAT1, RSAD2, MX2, OAS1&2, IFNL44 and LXN^43^. Finally, LXN, OAS1&2 and MX2 were observed to be upregulated in liver cirrhosis compared to normal liver^45^. Our finding that LXN overexpression potentially increases immune signaling, possibly through IFN-γ and its related genes is further supported by several studies that highlight the synergy between interferons and atRA in clearing infection^47,48^, and increasing apoptosis in cancer cells^49,50^.

Our new data describes LXN as a predominantly luminal protein that is downregulated in high grade primary and metastatic disease. In contrast to previous work within the prostate, we describe LXN protein as both cytosolic and secreted rather than predominantly nuclear. LXN did not appear to be silenced in normal prostate tissue stem cells and overexpression did not appear to affect epithelial basal cell fate as previously described. We demonstrate that LXN is directly and lastingly upregulated by atRA and show that ectopic overexpression indirectly drives a pro-inflammatory immunomodulatory gene signature.Taken together these data suggest that LXN may play a functional role as a secreted ligand in the inflammatory response^6,25^. For instance, LXN may be secreted by epithelial cells and influence immune cell differentiation, recruitment and/or activity. This would be in broad agreement with the LXN knockout mouse phenotype, which results in an over-representation of haematopoietic stem-like cells in bone marrow^6,7^. Therefore, loss of LXN expression by tumour cells might be a novel mechanism of tumour immune cell evasion, for instance by blocking maturation of B and T cells. This could provide a survival benefit *in vivo* by preventing adequate anti-tumour immune responses. Our findings therefore highlight the importance of future study into the effects of LXN on immune cell infiltration and function within the tumour micro-environment.

## Materials and Methods

We confirm that all research in this study was performed in accordance with local guidelines and (NHS Research Ethics Approval (REC) 07/H1304/121).

### Culture of Prostate Cell lines

PNT1A, PC3 and LNCaP cells were purchased from American Type Culture Collection (ATCC). P4E6 were derived in our laboratory^51^ and BPH1 were a kind gift from Dr Hayward (NorthShore University HealthSystem Research Institute). PNT1A and LNCaP were cultured in Roswell Park Memorial Institute medium (RPMI-1640) (Invitrogen Ltd) supplemented with 10% Foetal Calf Serum (FCS), and 2 mM L-Glutamine (Invitrogen Ltd). BPH1 cells were cultured in RPMI-1640 supplemented with 5% FCS, and 2 mM L-Glutamine. P4E6 cells were cultured in Keratinocyte Serum-Free Medium (KSFM) (Invitrogen Ltd) supplemented with 2% FCS, 2 mM L-Glutamine, 5 ng/ml Epidermal Growth Factor (EGF), 50 μg/ml bovine pituitary extract (Invitrogen Ltd). PC3 cells were cultured in Ham’s F-12 medium (Lonza) supplemented with 7% FCS and 2 mM L-Glutamine. All cell lines were cultured at low passage and routinely tested for *Mycoplasma* contamination (EZ-PCR kit; Geneflow).

### Primary cell culture

Primary cultures were derived from patient prostate tissues as described in^52^. Samples were obtained with informed written consent and ethical approval (NHS Research Ethics Approval (REC) 07/H1304/121). Tissue samples were provided along with histopathological analysis. Primary prostate epithelial cells were cultured in KSFM supplemented with 1% L-glutamine, 1 ng/ml GM-CSF, 2 ng/ml stem cell factor, 50 μg/ml bovine pituitary extract, 100 ng/ml cholera toxin, and 5 ng/ml epidermal growth factor on dishes pre-coated with collagen type 1. Early passage cultures were co-cultured with mitotically inactivated mouse embryonic fibroblast (STO) feeder cells. Cellular fractionation to enrich for α2β1hi/CD133+ (stem-like cells), α2β1hi/CD133− (transit amplifying, TA) and α2β1low (committed basal, CB) cells were conducted as previously described^19^. First adherence to collagen was used separate α2β1-hi and α2β1low cells. This was followed by MACS (Miltenyi Biotec Ltd.) selection using anti anti-human CD133 microbeads and selecting CD133+ cell as per the manufacturer’s instructions.

### Lentiviral transduction

Stable overexpression in prostate epithelial cells was achieved using lentiviral vectors. Human LXN cDNA was cloned into the lentiviral vectors, pDEST-236 (puromycin resistance vector) or pDEST-298 (eGFP co-expression vector). Lentiviral vectors using the Invitrogen Gateway Recombination Cloning Technology (Life Technologies Ltd). All lentiviral backbone vectors used in this study were a kind gift from Dr Stephan Geley. The pDONOR221-Escherichia coli-β-glucuronidase (Mock) was used for the control vector pGLTR-MOCK (Life Technologies Ltd). Cells were transduced with lentiviral particles and stable overexpression established using selection in puromycin or visualisation of eGFP positivity following standard protocols.

### Fluorescence activated cell sorting

Primary prostate tissue was digested as previously described^19^. Cells were harvested by layering the digestion supernatant onto lymphocyte separation media (MP biomedicals) and subsequent centrifugation. Epithelial cells were negatively enriched from stromal cells using magnetic labelled non-tumour cell depletion cocktail (Miltenyi Biotech). Resulting enriched epithelial cells were counted and stained with EPCAM-FITC, CD49b-APC and CD24-PE (Miltenyi Biotech), as per the manufacturer’s instructions. Epithelial cells were sorted on a MOFLO XDP using the following strategy: cells were gated from debris using FSC and SSC and live cells were gated based on Cytox blue negativity. Luminal Cells were sorted from the EPCAM+ CD24+ live cell population and Basal cells were sorted from the EPCAM+ CD24− CD49b+ cell populations. The non-epithelial, stromal cells were then enriched by purging the MACS column following magnetic labelling.

### Irradiation of cells

Cells were irradiated using an RS2000 X-Ray Biological Irradiator, containing a Comet MXR-165x-Ray Source (Rad-Source Technologies Inc., Suwanee, GA, USA). A dose of 60 Gy (STO cells) or 75 Gy (prostate cell lines) was administered with a dose rate of 0.08 Gy/s.

### Subcellular fractionation

Nucleocytoplasmic fractionation cell lines and primary cells was performed using NE-PER Nuclear and Cytoplasmic Extraction kit (Thermo Fisher Scientific) according to the manufacturer’s protocol.

### SDS-PAGE and Western blotting

Cell lysates were prepared in RIPA buffer as previously described^53^. Whole prostate tissue homogenates were prepared using TPER buffer (ThermoFisher scientific) according to the manufacturer’s instructions. Western blotting was performed as described previously^53,54^. Primary Antibodies used are listed in Supplementary Table S1. Western blots were cropped for use in figures using Adobe photoshop. Unedited Images are displayed in Supplementary Fig. S6–11.

### mRNA extraction

Total RNA was extracted using RNeasy Mini Kit (Qiagen) according to the manufacturer’s instructions. If STO cells were present in the culture, they were detached using 0.25% trypsin EDTA for 2 minutes at 37 °C. Determination of RNA quality and concentration was performed using a NanoDrop ND-1000 spectrophotometer (Thermo Fisher Scientific) or via a Bioanalyser (Agilent).

### cDNA Synthesis and Expression Analysis

Total RNA was reverse transcribed using Superscript III enzyme (Invitrogen) and random hexamers. RT-PCR reactions were performed using TaqMan gene expression assays and iTaq Universal Supermixes (Thermofiisher), according to the manufacturer’s protocol. The delta-delta Ct method was used to analyse the results of RT-PCR^55^. For relative quantification the target gene was normalised to the expression of a control gene (including RPLPO and 18S ribosomal RNA (18S). Affymetrix microarray analysis was performed by Eurofins using Affymetrix GeneChip® Human Transcriptome Array 2.0 and analysed using Transcriptome analysis software (TAC v4.0) (Invitrogen) according to standard protocols. A standardized cut-off of +/−2-fold change and a raw P value of <0.05 measured by 1-way ANOVA was used to select differentially expressed genes.

### Immunofluorescence

PNT1A Cells were fixed with 4% (w/v) paraformaldehyde pH 7.4 for 20 min prior to Permeabilisation using 0.5% (v/v) Triton X-100 for 25 min. After permeabilisation, cells were Blocked using with 10% (v/v) goat serum diluted 1% (w/v) BSA in PBS for 30 min. Antibody staining and slide preparation was then performed as previously described^53^. Analysis was performed using a Nikon Eclipse TE300 fluorescent microscope (Nikon) or LSM 720 meta confocal microscope (Zeiss). Primary antibody dilutions used for immunofluorescence are listed in Supplementary Table S2.

### Cell Viability Assay

The alamarBlue assay (Invitrogen) was used to assess cell viability according to manufacturer’s instructions, to control for the potential effects of LXN on plating efficiency, Viability reads were normalized to cell number using an automated cell counter.

### Clonogenic recovery Assay

Primary prostate epithelial cells were transduced as previously described. Cells were harvested by trypsinisation, counted, and plated at a density of 20 live cells/well in a 6-well culture plate in triplicate. Cells were co-cultured in the presence of irradiated STO cells. Culture medium was replaced every 48 hours. 14 days after plating, the plates were stained with crystal violet (PBS, 1% crystal violet, 10% ethanol). The number colonies as well as the number of cells in each colony were recorded.

### Plating efficiency

PNT1A, BPH-1, P4E6 and LNCaP cells were transduced as previously described. Cells were incubated on standard tissue culture plastic before non-adhered cells were removed through medium exchange. Plating efficiency was calculated using the alamarBlue assay to determine the number of adherent viable cells and results corroborated using an automated cell counter.

### Scratch assay (wound closure)

BPH-1 and PNT1A cells were transduced as previously described. Cells were plated at 80% confluency overnight. After 16 hours a wound was created using P1000 pipette tip. The width of wound was measured at relevant time points using Image J software. The average of 10 different measurements was taken and the relative percentage of wound closure was calculated relative to wound size at time point 0.

### Extracellular vesicle purification

Extracellular vesicles were isolated from cell-conditioned media by serial centrifugation. Cell-conditioned media was centrifuged at 400 g, 5 min, 2,000 g, 15 min, 10,000 g, 40 min and subsequently by centrifugation at 100,000 g (Optima-MAX ultracentrifuge, with TLA110 rotor and Optiseal tubes, Beckman coulter) for 60 min to pellet vesicles. Alternatively, pre-cleared conditioned medium was overlaid onto a 30% sucrose, D2O cushion, and subject to centrifugation in a swing out rotor (MLS50, Beckman Coulter) for 90 mins. The collected sucrose was diluted in excess PBS, and vesicles were pelleted as above. This produced two experimental resources, a final pellet (of extracellular vesicles) and a supernatant (vesicle depleted media)^56^. Nano particle tracking analysis was employed to estimate the size of the vehicles purified using this methodology as we described^56^.

### Protein Half-life Assay

BPH-1 cells were cultured in the presence of 10 µM anisomycin (Sigma-Aldrich) for 0, 2, 4, 8, 16 or 24 hours. Protein lysates were prepared in RIPA buffer. The amount of LXN and p53 protein was assessed using western Blot as previously described.

### atRA treatment

Cells were cultured in the presence of atRA (Sigma-Aldrich) (typically 500 nM or 1 µM). Cells were harvested after treatment, time points ranged from 30 minutes to 96 hours depending on the experimental requirements. Induction of LXN was analysed using RT-PCR and western blot. HOXA1 was used as a positive control for atRA mediated upregulation.

### ApoTox-Glo™ Triplex Assay

Cell viability, cytotoxicity and apoptosis were assessed using the ApoTox-Glo™ Triplex Assay (Promega) according to the manufacturer’s instruction. Measurements were taken using a microplate reader (Polarstar Optima). Viability was measured at 505 nm (excitation at 400 nm). Cytotoxicity was measured 520 nm (excitation at 485 nm). Apoptosis was measured using luminescence.

### Statistical analysis

All statistics were performed using the Graphpad Prism 7.0 software. Data is presented as mean and standard error of the mean (SEM) unless otherwise stated. To test the significance between 2 data sets we used the Man-whitney test. When comparing more than 2 means, ANOVA was used, unless otherwise stated.

## Supplementary information

Supplementary information

## Data Availability

The datasets generated during or analysed during the current study are available from the corresponding author on reasonable request.
