# Supplementary material for: The putative tumour suppressor protein Latexin is secreted by prostate luminal cells and is downregulated in malignancy
Source: Sci Rep. 2019 Mar 26;9:5120. doi: 10.1038/s41598-019-41379-8 (PMC6435711; doi:10.1038/s41598-019-41379-8)
Supplement: Supplementary file 1 — Supplementary information [file 41598_2019_41379_MOESM1_ESM.pdf]

The putative tumour suppressor protein Latexin is secreted by prostate luminal cells and is downregulated in malignancy

Robert I. Seed[1†*], Alberto J. Taurozzi[1†] Daniel Wilcock[1†], Giovanna Nappo[1], Holger, H, H. Erb[1] Martin L. Read[3], Mark Gurney[5], Leanne K. Archer[1], Saburo Ito[6] Martin G. Rumsby[1], John L. Petrie[2], Aled Clayton[4,] Norman J. Maitland[1#] and Anne T. Collins[1#]

1. Cancer research Unit, Department of Biology, University of York, Heslington, York YO10 5DD
2. Department of Biology, University of York. University of York, Heslington, York YO10 5DD
3. Institute of Metabolism and Systems Research,College of Medical and Dental Sciences, University of Birmingham, Edgbaston, Birmingham, B15 2TT
4. Tissue Microenvironment Group, Division of Cancer and Genetics, Tenovus Building, Heath Park, Cardiff University CF14 4XN
5. Division of Infection and Immunity, School of Medicine, Cardiff University, Heath Park, Cardiff CF14 4XN
6. Department of Pathology, University of California San Francisco, San Francisco, CA 94110

[†]These authors contributed equally
# joint senior authors

*Corresponding author: Robertseed@outlook.com Robert.seed@ucsf.edu

Supplementary methods

siRNA Knockdown

Cell lines were transfected with siRNAs *LXN* (siRNA ID: s230651 or s32409) or negative control #1(Ambion) at a final concentration of 50 nM using Lipofectamine RNAiMAX (Thermo Fisher Scientific, Waltham, MA). as transfection reagent as per the manufacturer's instructions

Transfection of Cell Lines

ViaFect transfection reagent (Promega) was used to transfect cell lines with plasmid DNA according to the manufacturer's instructions. Each transfection reaction consisted of 100-2500 ng plasmid DNA diluted in OptiMEM (GIBCO) serum-free medium. Cells were incubated in the presence of plasmid-ViaFect complexes overnight at 37°C. After incubation the DNA- ViaFect complexes were aspirated and replaced with fresh culture medium.

Assessing viral integration

A qPCR assay using primers specific to the woodchuck hepatitis virus posttranscriptional regulatory element (WPRE) was used to estimate viral integration. WPRE is found on all destination vectors used in this study and is integrated along with the transgene. The assay also incorporated primers specific for the single copy RNase P gene. DNA was prepared using phenol-chloroform extraction. The DNA extractions were interrogated with both the WPRE and

RNase P primers along with a 5 point standard curve for each set of primers. Integrations per cell is calculated as follows:

Integrations per cell = WPRE copy number ÷ (RNase P copy number ÷ 2)

Analysis of exosomal fractions

A continuous sucrose gradient (0.2 to 2.5M) was poured, and pre-concentrated vesicles overlaid before an overnight centrifugation at 210,000g as previously described[1]. Collected fractions were washed in PBS by pelleting and assayed for vesicle markers performed by plate assay. Briefly, fractions were immobilized on the plates overnight in PBS and blocked for 2 h in 1% (w/v) BSA/PBS. Wells were incubated in anti-CD81 antibody at 2ug/ml (Serotec, Oxford, UK). Detection was by goat anti-mouse biotinylated antibodies (PerkinElmer Life). To assess signal, we added Europium-streptavidin conjugate and, following six washes, measured it via time-resolved fluorimetry on a Wallac Victor-II multi-label plate reader (PerkinElmer Life)[2]

Gene ontology analysis

Gene ontology was performed using the PANTHER overrepresentation analysis tool. Statistical analysis was performed using Fisher's exact test and Bonferroni correction. The top 256 differentially expressed genes were assessed against the homo sapiens database.

Supplementary references

1. Welton, J. L. *et al.* Proteomics analysis of bladder cancer exosomes. *Mol. Cell. Proteomics MCP* **9**, 1324–1338 (2010).
2. Webber, J. *et al.* Proteomics Analysis of Cancer Exosomes Using a Novel Modified Aptamer-based Array (SOMAscanTM) Platform. *Mol. Cell. Proteomics MCP* **13**, 1050–1064 (2014).

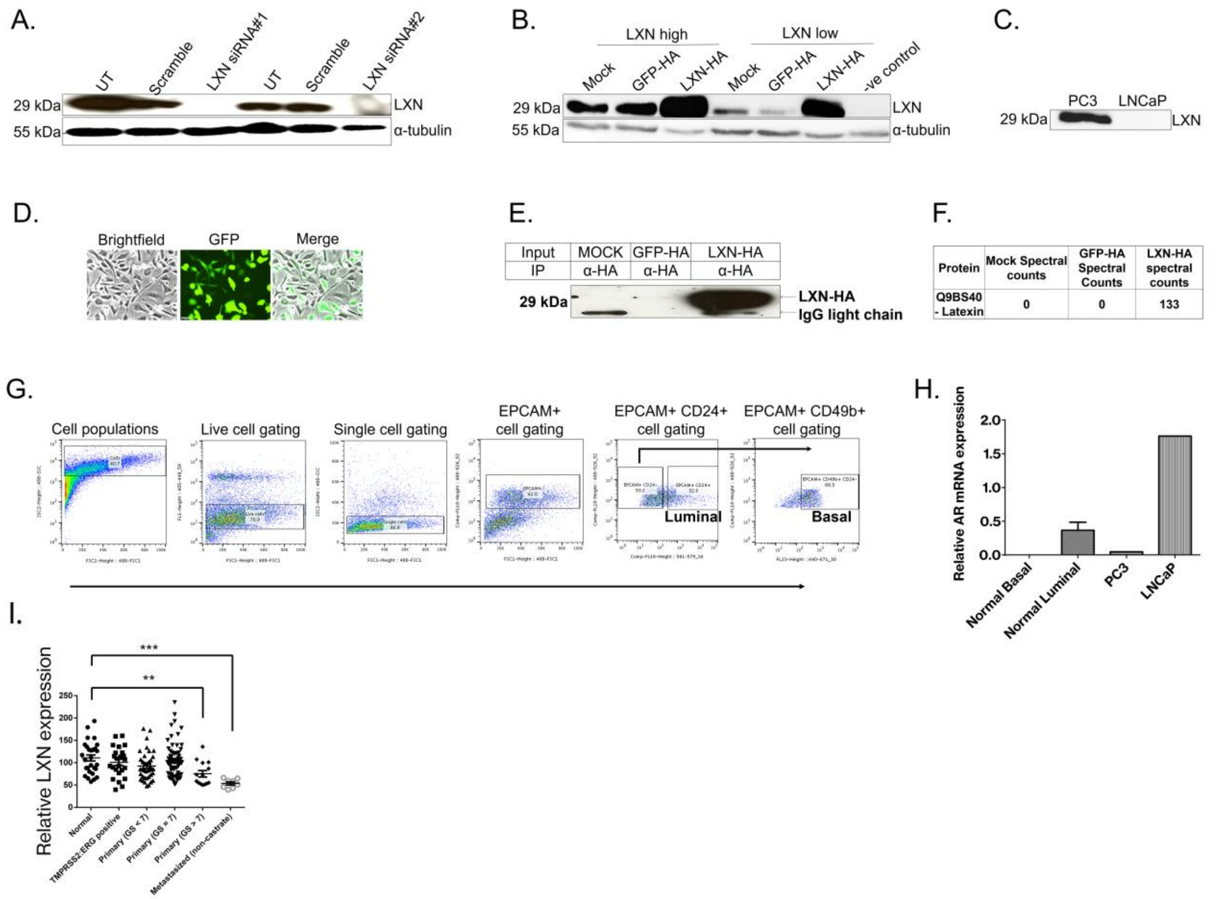

Supplementary Figure S1. Validation of a LXN-specific antibody. **A**. Western blot, demonstrating a single band of predicted size, in PC3 cells, and loss of LXN expression 72 hours after knockdown using two specific LXN siRNAs. **B**. Western blot demonstrating LXN overexpression 48 hours after transfection of a LXN expression vector compared to transfection controls, in LXN high and low expressing cell lines respectively. Included is the LXN non-expressing cell line LNCaP. **C**. The specificity of the antibody is further confirmed by comparison of LXN protein expression from PC3 (high expressing) and LNCaP (non-expressing) cells respectively. **D&E** Transient overexpression of HA-tagged LXN in PC3 cells can be detected following immunoprecipitation of LXN-HA using a highly specific anti-HA antibody and subsequent detection of the IP product using the anti LXN antibody. **F**. Mass spectrometry analysis of the IP product from **E** revealed an abundance of LXN compared to controls. **G**. A schematic to demonstrate the cell sorting strategy to enrich for basal and luminal cells from primary prostate epithelial tissue. Cells were gated and assessed for viability using Cytox blue, before gating single cell populations. EPCAM positivity and CD24 positivity were used to sort enriched luminal cell populations, and $EPCAM^+$, $CD24^-$ $CD49b^+$ populations were used to sort enriched basal epithelial cell populations. **H**. mRNA expression of the luminal specific marker AR confirms the efficacy of our FACS sorting approach (PC3 basal-type cells and LNCap luminal-type cells serve as negative and positive controls respectively). **I.** Stratification of patient samples by Gleason score reveals that LXN is downregulated in cancers graded greater than Gleason 7.

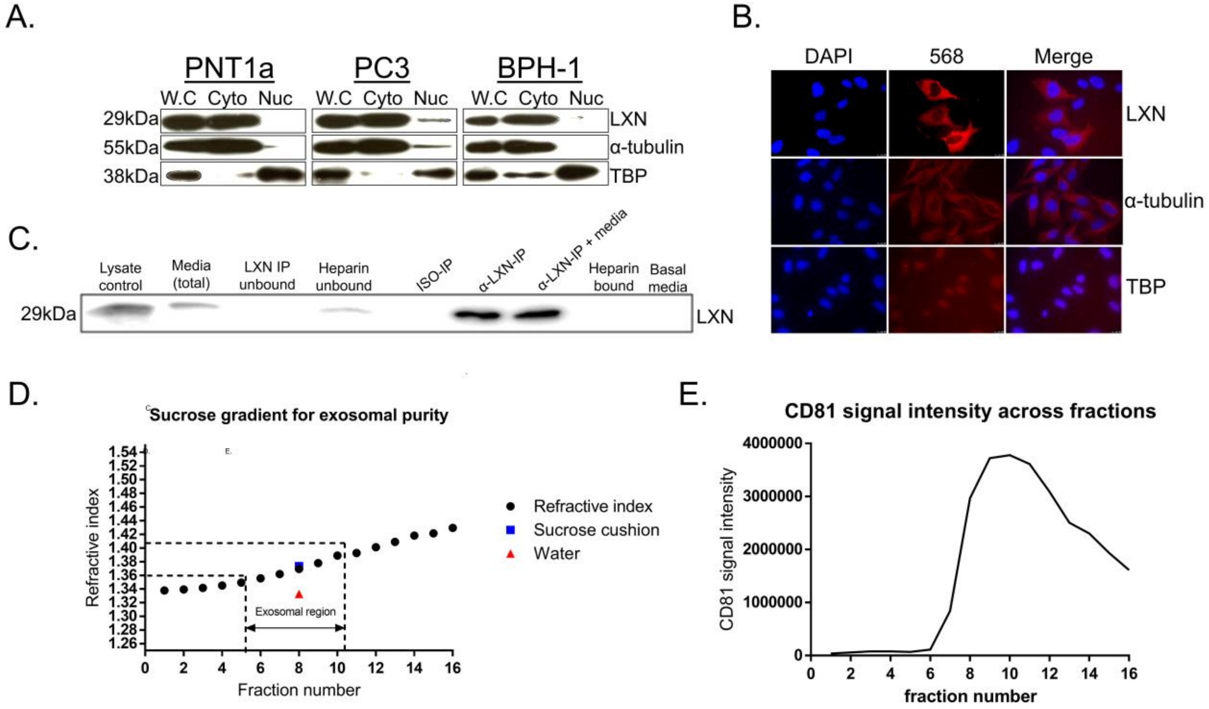

Supplementary Figure S2. **A**. Overexposed WB of whole cell (W.C), cytosolic (cyto) and nuclear (nuc) extracts in PNT1a, PC3 and BPH-1 cells. α –tubulin and TBP are cytosolic and nuclear markers respectively **B**. Immunocytochemistry of PNT1a cells following transient overexpression of LXN-HA (upper panel). α-tubulin (middle panel) and TBP (lower panel) demonstrate the unbiased accessibility of both the nuclear and cytosolic compartments using this approach **C**. Full length blot from Fig 2E showing Immunoprecipitation of LXN using the latexin specific antibody (LXN-IP) from PNT1a cell culture conditioned media compared to an isotype matched negative control (ISO-IP). In addition, binding to heparin was assessed by incubation with heparin agarose beads, a control containing only basal media serves to demonstrate that LXN does not bind to Bovine LXN potentially present in serum containing media. **D**. A sucrose cushion was generated to purify exosomes EVs and was found to be consistent with the RI required to purify Evs. **E**. ELISA-based assay of CD81 expression across fractions confirms that the sucrose cushion adequately captures an abundance of EVs, CD81 is a marker of EVs.

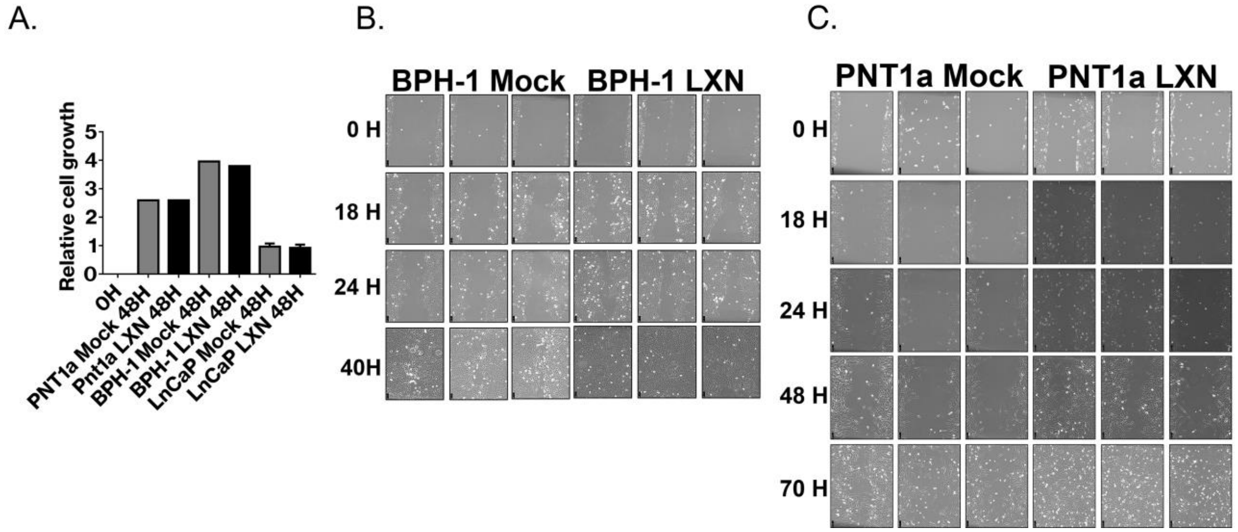

Supplementary Figure S3. **A**. Cell proliferation assays depicting the growth of PNT1a, BPH-1 or LNCaP cells stably overexpressing LXN compared to (mock) controls measured via the cell counting method. **B&C**. Representative images used to calculate the effects of LXN overexpression on relative wound closure over time in BPH-1 and PNT1a cells.

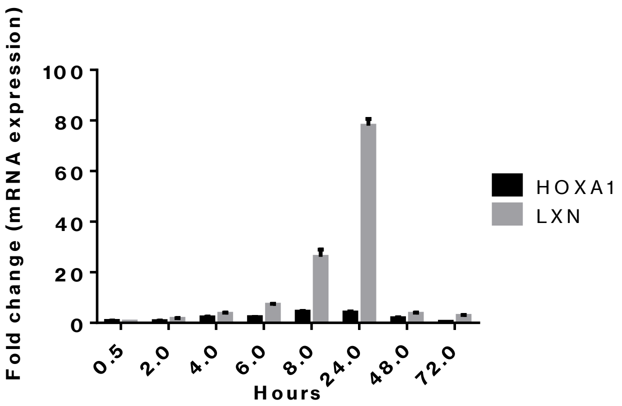

Supplementary Figure S4. 72 hours time-course demonstrating the induction of LXN and HOXa1 mRNA expression in PNT1a cells following treatment with 1 µM atRA.

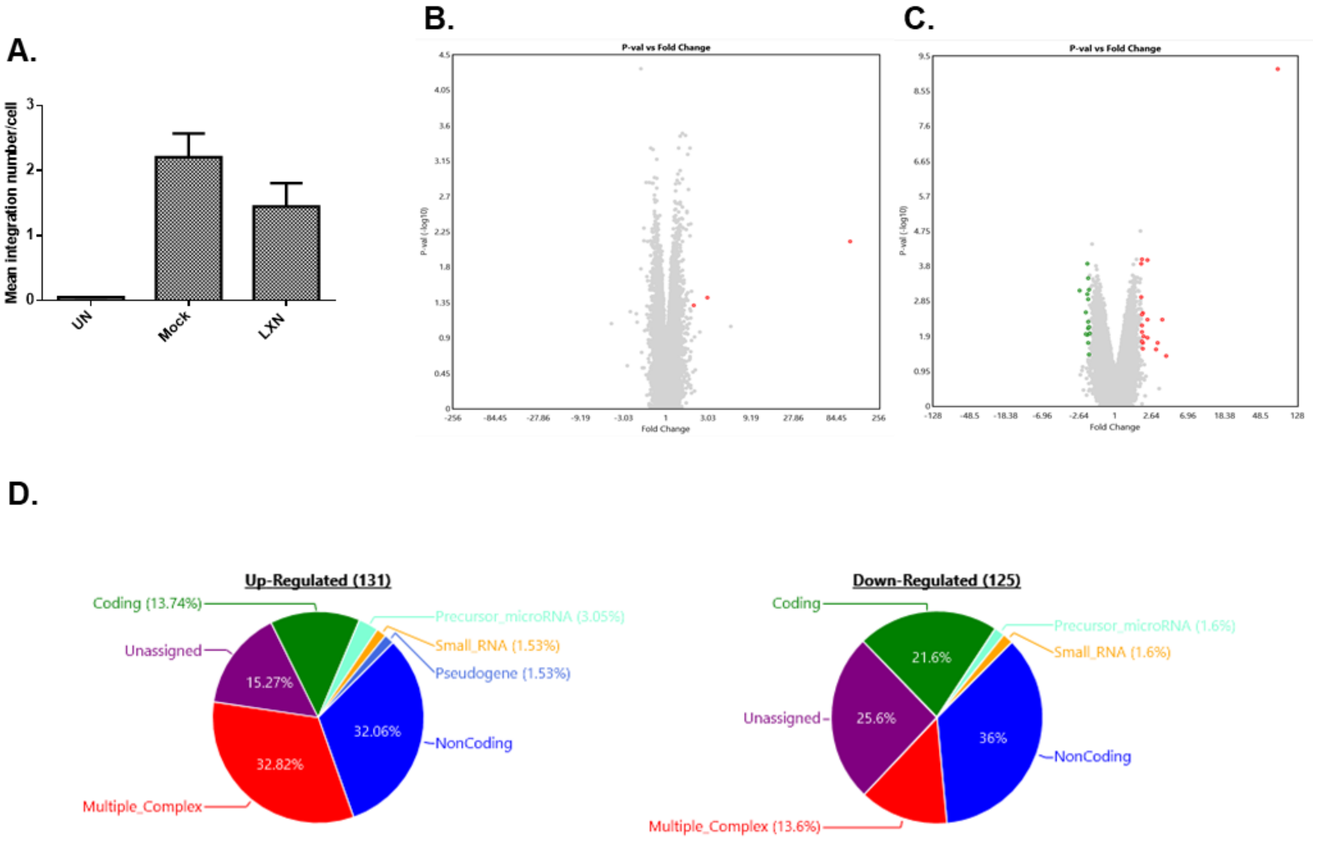

Supplementary Figure S5. **A**. PCR data demonstrating that the mean viral integration number obtained following transduction of primary human prostate epithelia is consistent between Mock and LXN viral titres as expected. Integration is measured using the viral woodchuck gene which integrates along with the transgene **B**. Representative volcano plot depicting differentially expressed genes in response to short-term overexpression (cut off was ±2-fold change *p<0.05).* **C**. Representative volcano plot demonstrating the number of differentially expressed genes following long-term overexpression of LXN in primary prostate epithelia (cut off was ±2-fold change *p<0.05).* **D**. Long-term/stable overexpression of LXN resulted in differential expression of genes compared to mock controls. Pie chart depicting the types of genes altered due to LXN expression. Of 13570 genes identified, 256 (0.19%) passed the criteria for selection (<-2 or >2 fold p<0.05) where 131 (51.17%) were found to be up-regulated and 125 (48.83%) to be down-regulated in response to stable LXN overexpression.

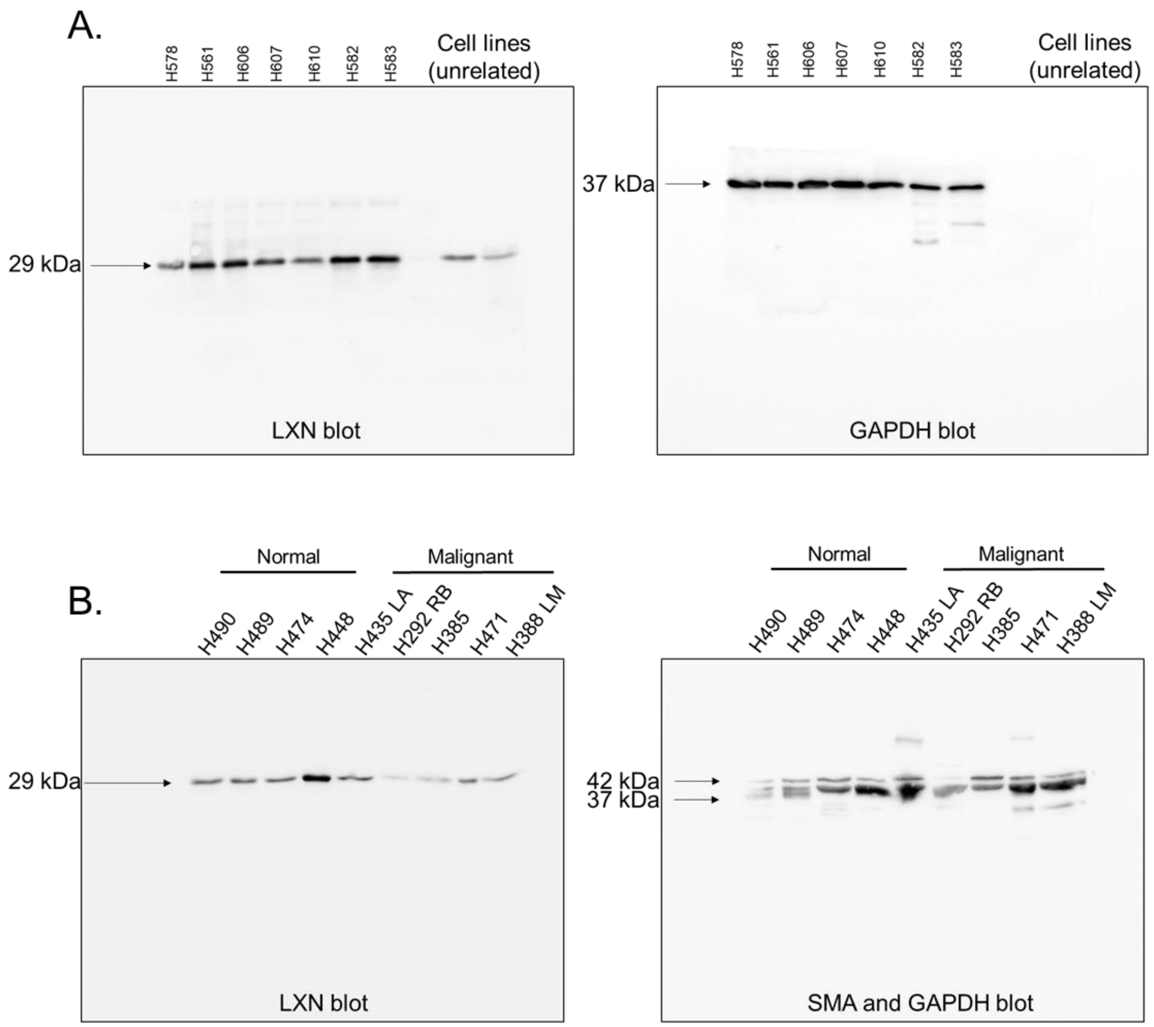

Supplementary Figure S6: A. Full length uncropped WB images depicting LXN and GAPDH expression from Fig 1A. Note that 3 unrelated samples are present on the right of the WB image. **B.** Full length uncropped WB images depicting LXN, α-SMA and GAPDH expression From Fig 1E.

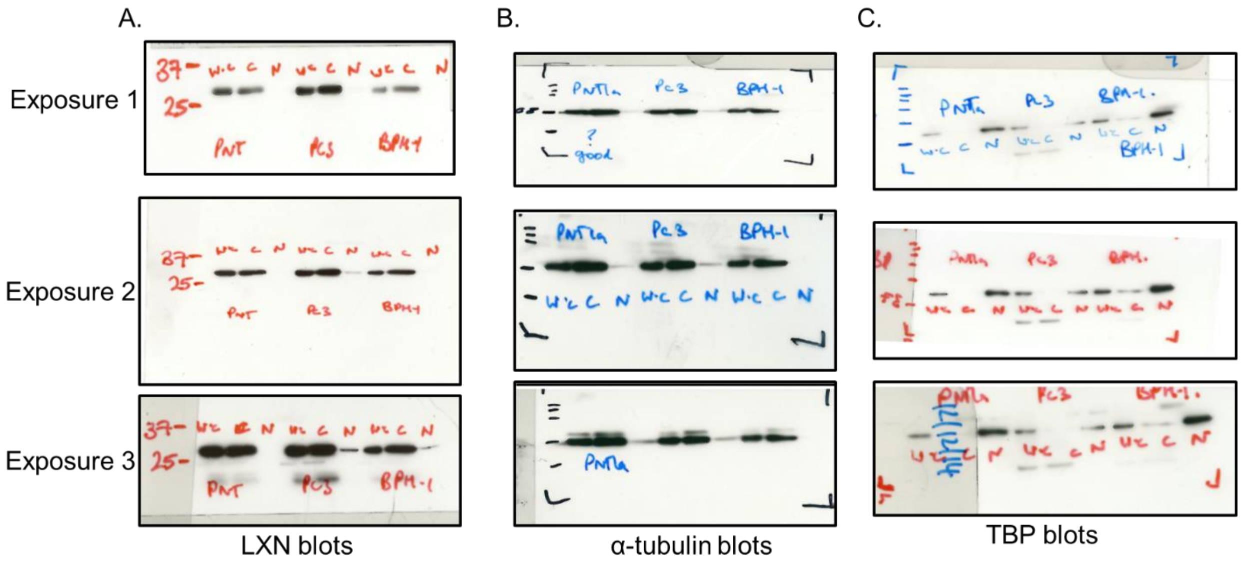

Supplementary Figure S7: Full length uncropped WB images depicting LXN **(A)**, α-tubulin **(B)**, or TBP **(C)**, at 3 different exposures. Images from exposure 2 were used to generate Fig 2A.

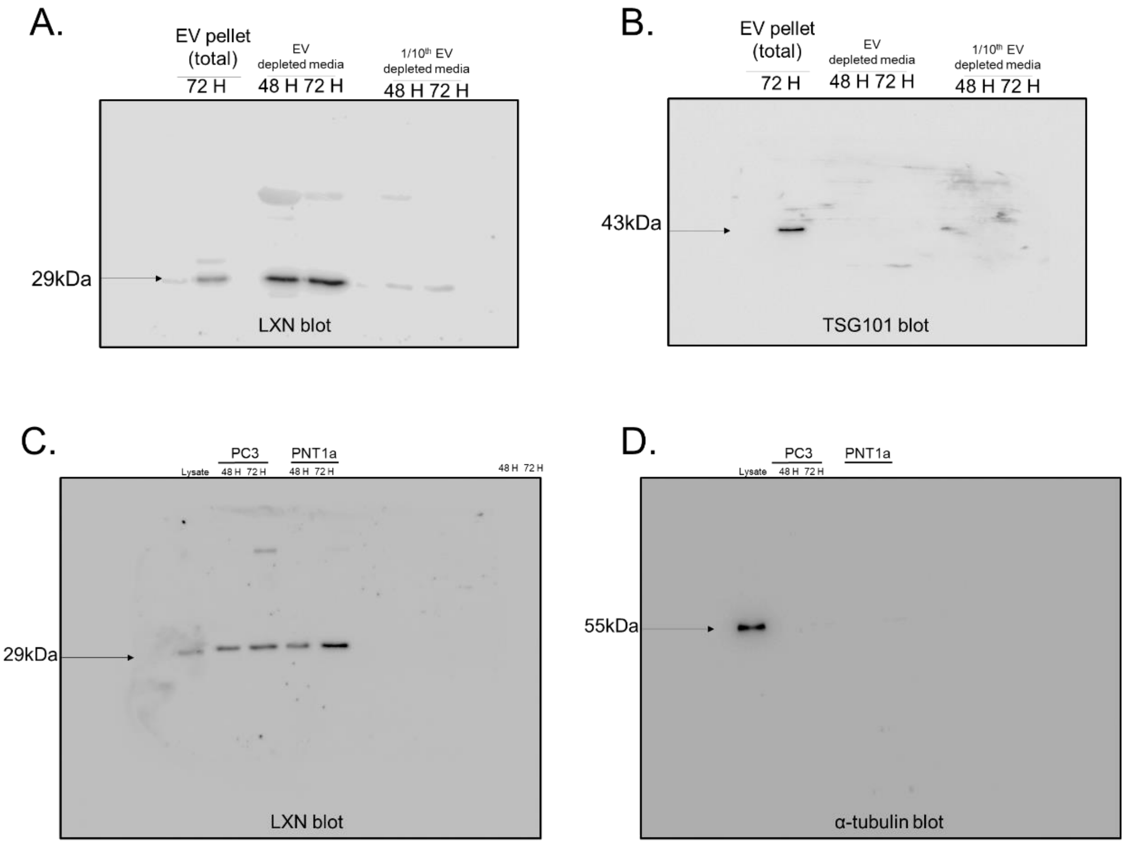

Supplementary Figure S8. Full length uncropped WB images depicting LXN **(A)**, TSG101 **(B)**, LXN **(C)** and α-tubulin **(D)**. Images were used to generate Fig 2K&L respectively.

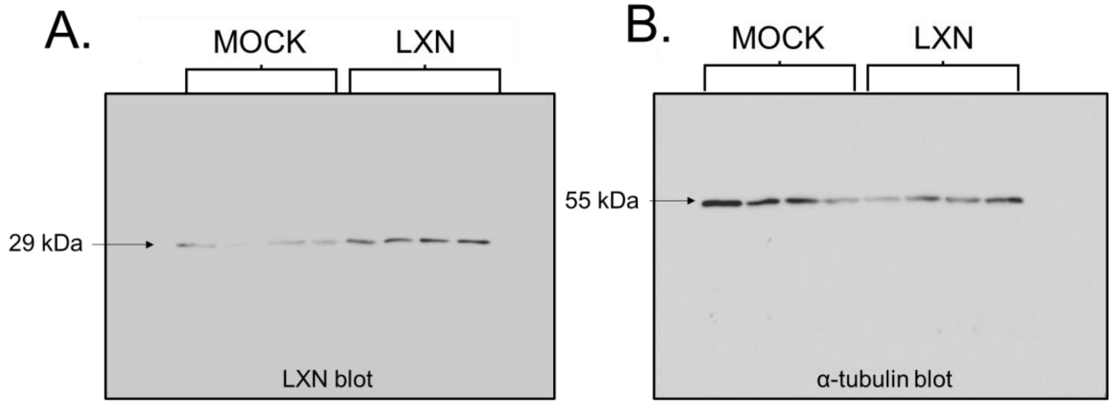

Supplementary Figure S9: Full length uncropped WB images depicting LXN **(A)** and α-tubulin **(B)**. Images were used to generate Fig 3A.

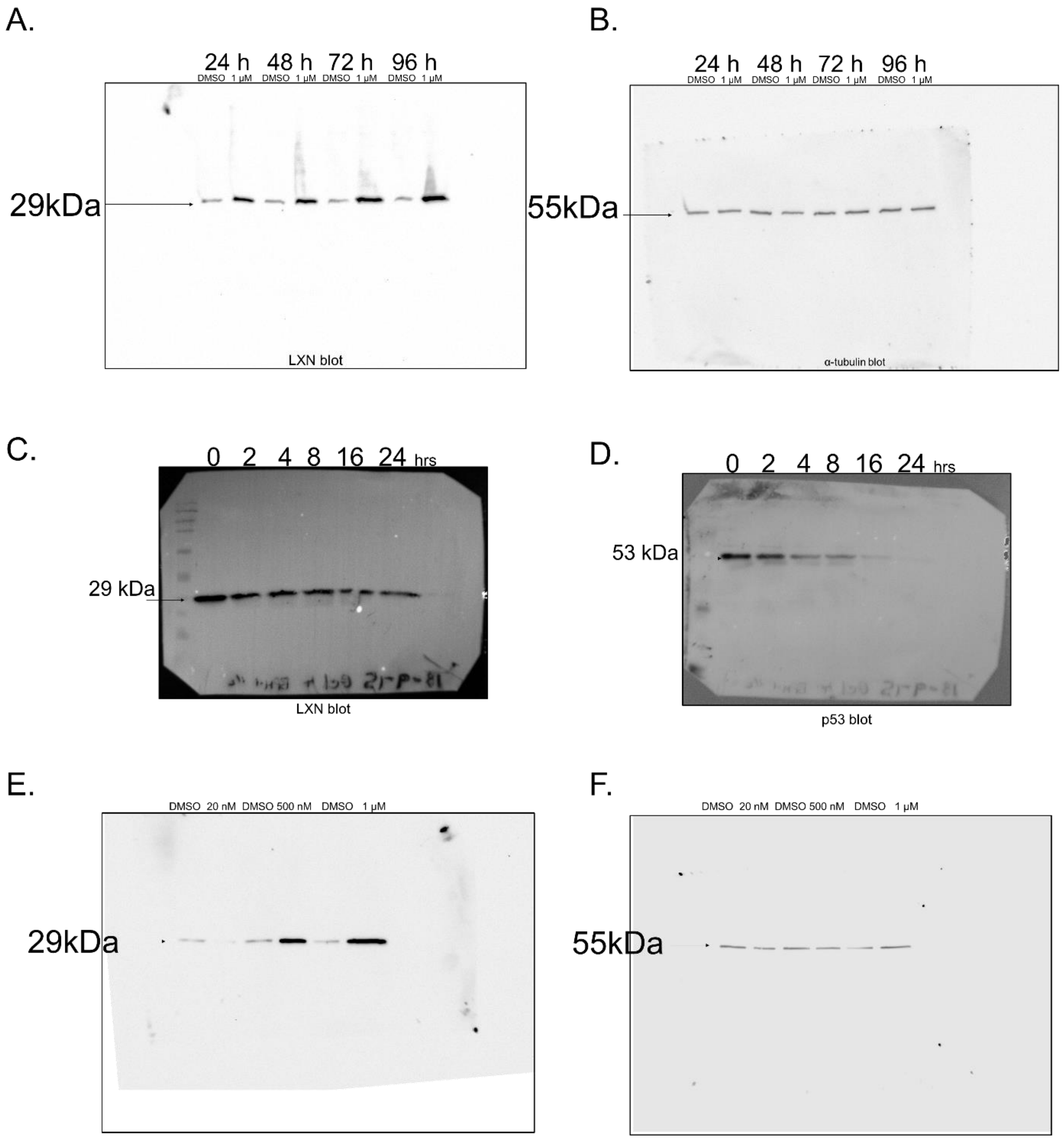

Supplementary Figure S10: Full length uncropped WB images used to generate Fig 4B **(A&B)**, Fig 4C **(C&D)** and Fig 4D **(E&F)**.

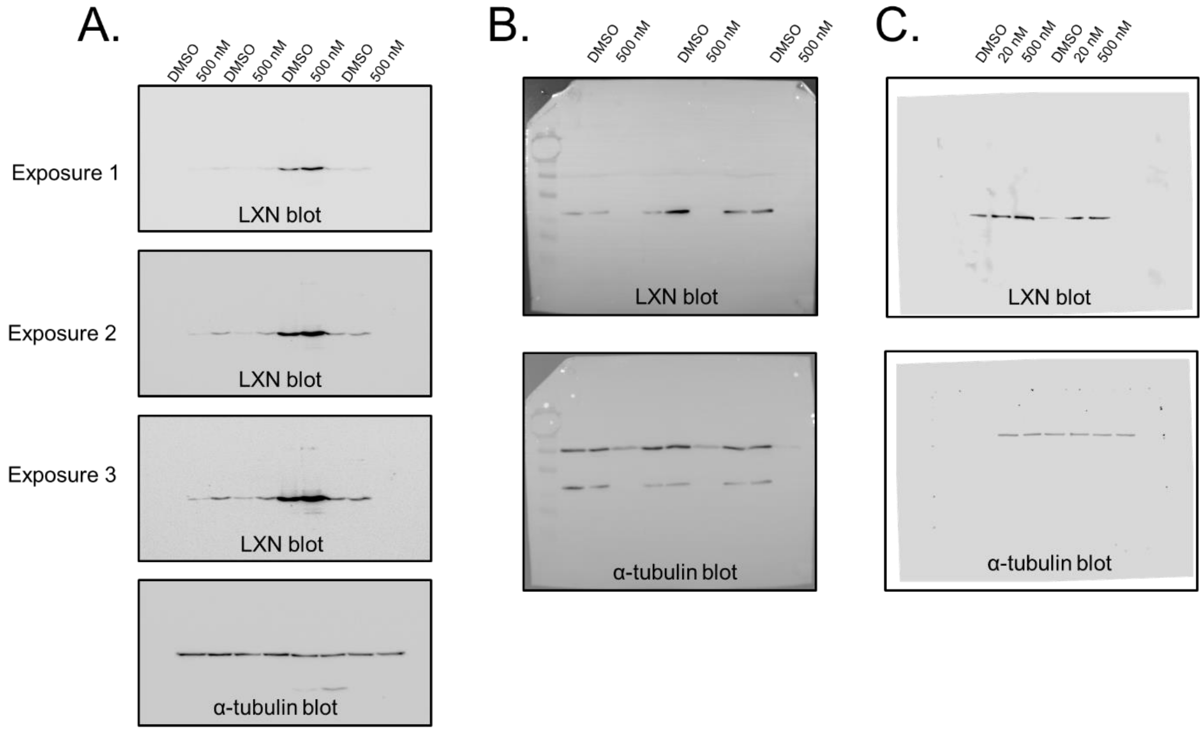

Supplementary Figure S11: Full length uncropped WB images used to generate Fig 4G. **A.** multiple LXN exposures form the same blot with accompanying α-tubulin loading control. **B.** Full length LXN and α-tubulin blot respectively. **C.** length LXN and α-tubulin blot respectively

Supplementary table S1: Antibodies used in this study

Antibody	Product no.	Clone	Western Blot	Immunocytochemistry
LXN	AB154744	Polyclonal	0.3 ug/ml	N/A
α-tubulin	AB7921	Monoclonal [DM1A]	1:1000	1:200
TATA-BP	AB818	Monoclonal [1TBP18]	1:1000	1:200
GAPDH	AB8245	monoclonal [6C5]	1:3000	N/A
Smooth muscle actin	A2547	Monoclonal [1A4]	1:1000	N/A
TSG101	sc-7964	Polyclonal	1:1000	N/A
TP53 (total)	sc-126	polyclonal [DO-1]	1:1000	N/A
HA	AB18181	Monoclonal [HA.C5]	1:1000	1:500

Supplementary table S2 complete gene ontology terms for differentially expressed genes following long-term overexpression of LXN in primary prostate epithelial cells.

GO biological process complete	Homo sapiens - REFLIST (20996)	upload_1 (254)	upload_1 (expected)	upload_1 (over/under)	upload_1 (fold Enrichment)	upload_1 (P-value)
**regulation of granulocyte differentiation (GO:0030852)**	17	5	0.21	+	24.31	4.56E-02
**regulation of mast cell degranulation (GO:0043304)**	28	6	0.34	+	17.71	2.44E-02
**regulation of mast cell activation involved in immune response (GO:0033006)**	29	6	0.35	+	17.1	2.91E-02
**regulation of mast cell activation (GO:0033003)**	39	7	0.47	+	14.84	1.01E-02
**regulation of myeloid leukocyte mediated immunity (GO:0002886)**	52	9	0.63	+	14.31	3.80E-04
**regulation of leukocyte degranulation (GO:0043300)**	42	7	0.51	+	13.78	1.57E-02
**negative regulation of tumor necrosis factor superfamily cytokine production (GO:1903556)**	55	8	0.67	+	12.02	7.15E-03
**regulation of interleukin-1 beta production (GO:0032651)**	66	8	0.8	+	10.02	2.48E-02
**regulation of chemokine production (GO:0032642)**	75	9	0.91	+	9.92	6.36E-03
**regulation of interleukin-1 production (GO:0032652)**	80	9	0.97	+	9.3	1.04E-02

**positive regulation of lymphocyte differentiation (GO:0045621)**	93	10	1.13	+		8.89	3.87E-03
**regulation of leukocyte chemotaxis (GO:0002688)**	109	10	1.32	+		7.58	1.48E-02
**myeloid leukocyte migration (GO:0097529)**	124	11	1.5	+		7.33	6.07E-03
**regulation of viral life cycle (GO:1903900)**	139	12	1.68	+		7.14	2.47E-03
**regulation of leukocyte mediated immunity (GO:0002703)**	198	17	2.4	+		7.1	8.49E-06
**positive regulation of leukocyte differentiation (GO:1902107)**	143	12	1.73	+		6.94	3.28E-03
**regulation of tumor necrosis factor production (GO:0032680)**	133	11	1.61	+		6.84	1.15E-02
**regulation of tumor necrosis factor superfamily cytokine production (GO:1903555)**	137	11	1.66	+		6.64	1.51E-02
**regulation of leukocyte migration (GO:0002685)**	182	14	2.2	+		6.36	9.46E-04
**response to interferon-gamma (GO:0034341)**	183	14	2.21	+		6.32	1.01E-03
**cellular response to lipopolysaccharide (GO:0071222)**	185	14	2.24	+		6.26	1.14E-03
**cellular response to interferon-gamma (GO:0071346)**	161	12	1.95	+		6.16	1.06E-02

**regulation of lymphocyte mediated immunity (GO:0002706)**	148	11	1.79	+	6.14	3.04E-02
**regulation of lymphocyte differentiation (GO:0045619)**	164	12	1.98	+	6.05	1.27E-02
**cellular response to molecule of bacterial origin (GO:0071219)**	192	14	2.32	+	6.03	1.75E-03
**regulation of response to cytokine stimulus (GO:0060759)**	166	12	2.01	+	5.98	1.43E-02
**regulation of leukocyte proliferation (GO:0070663)**	222	16	2.69	+	5.96	2.70E-04
**positive regulation of hemopoiesis (GO:1903708)**	182	13	2.2	+	5.9	5.97E-03
**regulation of cytokine-mediated signaling pathway (GO:0001959)**	156	11	1.89	+	5.83	4.89E-02
**regulation of mononuclear cell proliferation (GO:0032944)**	214	15	2.59	+	5.79	1.03E-03
**regulation of cytokine secretion (GO:0050707)**	191	13	2.31	+	5.63	9.95E-03
**regulation of viral process (GO:0050792)**	177	12	2.14	+	5.6	2.68E-02
**negative regulation of defense response (GO:0031348)**	193	13	2.33	+	5.57	1.11E-02
**positive regulation of immune effector process (GO:0002699)**	209	14	2.53	+	5.54	4.59E-03
**regulation of lymphocyte**	213	14	2.58	+	5.43	5.69E-03

**proliferation (GO:0050670)**						
**positive regulation of I-kappaB kinase/NF-kappaB signaling (GO:0043123)**	183	12	2.21	+	5.42	3.71E-02
**cellular response to biotic stimulus (GO:0071216)**	214	14	2.59	+	5.41	6.00E-03
**positive regulation of leukocyte cell-cell adhesion (GO:1903039)**	216	14	2.61	+	5.36	6.66E-03
**regulation of immune effector process (GO:0002697)**	451	29	5.46	+	5.32	7.51E-09
**positive regulation of T cell activation (GO:0050870)**	203	13	2.46	+	5.29	1.88E-02
**regulation of symbiosis, encompassing mutualism through parasitism (GO:0043903)**	206	13	2.49	+	5.22	2.20E-02
**regulation of chemotaxis (GO:0050920)**	206	13	2.49	+	5.22	2.20E-02
**positive regulation of cell-cell adhesion (GO:0022409)**	254	16	3.07	+	5.21	1.54E-03
**myeloid cell differentiation (GO:0030099)**	210	13	2.54	+	5.12	2.68E-02
**negative regulation of cytokine production (GO:0001818)**	260	16	3.15	+	5.09	2.07E-03
**regulation of T cell activation (GO:0050863)**	312	19	3.77	+	5.03	1.75E-04
**regulation of I-kappaB kinase/NF-**	231	14	2.79	+	5.01	1.41E-02

**kappaB signaling (GO:0043122)**						
**regulation of cytokine production (GO:0001817)**	661	40	8	+	5	1.56E-12
**regulation of leukocyte differentiation (GO:1902105)**	269	16	3.25	+	4.92	3.19E-03
**response to molecule of bacterial origin (GO:0002237)**	327	19	3.96	+	4.8	3.55E-04
**response to lipopolysaccharide (GO:0032496)**	313	18	3.79	+	4.75	9.44E-04
**negative regulation of response to external stimulus (GO:0032102)**	313	18	3.79	+	4.75	9.44E-04
**negative regulation of cell adhesion (GO:0007162)**	262	15	3.17	+	4.73	1.17E-02
**regulation of inflammatory response (GO:0050727)**	317	18	3.83	+	4.69	1.13E-03
**regulation of innate immune response (GO:0045088)**	375	21	4.54	+	4.63	1.23E-04
**negative regulation of immune system process (GO:0002683)**	429	24	5.19	+	4.62	1.11E-05
**regulation of leukocyte activation (GO:0002694)**	579	32	7	+	4.57	2.13E-08
**positive regulation of cytokine production (GO:0001819)**	419	23	5.07	+	4.54	3.50E-05

**regulation of leukocyte cell-cell adhesion (GO:1903037)**	293	16	3.54	+		4.51	9.34E-03
**myeloid leukocyte mediated immunity (GO:0002444)**	515	28	6.23	+		4.49	7.92E-07
**regulation of response to external stimulus (GO:0032101)**	744	40	9	+		4.44	6.67E-11
**regulation of defense response (GO:0031347)**	670	36	8.11	+		4.44	1.75E-09
**positive regulation of response to external stimulus (GO:0032103)**	298	16	3.61	+		4.44	1.15E-02
**regulation of cell activation (GO:0050865)**	616	33	7.45	+		4.43	2.10E-08
**regulation of lymphocyte activation (GO:0051249)**	504	27	6.1	+		4.43	2.40E-06
**leukocyte degranulation (GO:0043299)**	505	27	6.11	+		4.42	2.50E-06
**neutrophil mediated immunity (GO:0002446)**	494	26	5.98	+		4.35	7.48E-06
**innate immune response (GO:0045087)**	741	39	8.96	+		4.35	2.89E-10
**neutrophil activation (GO:0042119)**	495	26	5.99	+		4.34	7.79E-06
**inflammatory response (GO:0006954)**	477	25	5.77	+		4.33	1.75E-05
**positive regulation of cell adhesion (GO:0045785)**	403	21	4.88	+		4.31	3.98E-04

**granulocyte activation (GO:0036230)**	500	26	6.05	+	4.3	9.53E-06
**neutrophil degranulation (GO:0043312)**	483	25	5.84	+	4.28	2.22E-05
**regulation of cell-cell adhesion (GO:0022407)**	389	20	4.71	+	4.25	1.04E-03
**neutrophil activation involved in immune response (GO:0002283)**	487	25	5.89	+	4.24	2.60E-05
**positive regulation of defense response (GO:0031349)**	390	20	4.72	+	4.24	1.08E-03
**positive regulation of cell activation (GO:0050867)**	410	21	4.96	+	4.23	5.25E-04
**myeloid leukocyte activation (GO:0002274)**	572	29	6.92	+	4.19	1.71E-06
**positive regulation of leukocyte activation (GO:0002696)**	398	20	4.81	+	4.15	1.47E-03
**myeloid cell activation involved in immune response (GO:0002275)**	518	26	6.27	+	4.15	1.93E-05
**positive regulation of lymphocyte activation (GO:0051251)**	359	18	4.34	+	4.14	6.39E-03
**cellular response to lipid (GO:0071396)**	524	26	6.34	+	4.1	2.43E-05
**leukocyte migration (GO:0050900)**	365	18	4.42	+	4.08	8.02E-03
**regulation of cell adhesion (GO:0030155)**	665	32	8.04	+	3.98	6.52E-07

**regulation of multi-organism process (GO:0043900)**	377	18	4.56	+	3.95	1.25E-02
**positive regulation of immune system process (GO:0002684)**	1064	50	12.87	+	3.88	3.08E-12
**regulation of endopeptidase activity (GO:0052548)**	409	19	4.95	+	3.84	9.33E-03
**regulated exocytosis (GO:0045055)**	693	32	8.38	+	3.82	1.77E-06
**regulation of hemopoiesis (GO:1903706)**	397	18	4.8	+	3.75	2.50E-02
**leukocyte mediated immunity (GO:0002443)**	759	34	9.18	+	3.7	9.81E-07
**regulation of immune response (GO:0050776)**	1056	47	12.78	+	3.68	1.92E-10
**leukocyte activation (GO:0045321)**	900	40	10.89	+	3.67	2.28E-08
**leukocyte activation involved in immune response (GO:0002366)**	613	27	7.42	+	3.64	1.32E-04
**defense response (GO:0006952)**	1302	57	15.75	+	3.62	3.63E-13
**regulation of peptidase activity (GO:0052547)**	434	19	5.25	+	3.62	2.15E-02
**cell activation involved in immune response (GO:0002263)**	617	27	7.46	+	3.62	1.50E-04
**positive regulation of immune response (GO:0050778)**	778	34	9.41	+	3.61	1.83E-06
**regulation of immune system**	1557	68	18.84	+	3.61	1.57E-16

**process (GO:0002682)**						
**exocytosis (GO:0006887)**	781	34	9.45	+	3.6	2.02E-06
**immune effector process (GO:0002252)**	1070	46	12.94	+	3.55	1.26E-09
**regulation of cell migration (GO:0030334)**	816	35	9.87	+	3.55	1.56E-06
**cell-cell adhesion (GO:0098609)**	492	21	5.95	+	3.53	9.24E-03
**response to bacterium (GO:0009617)**	680	29	8.23	+	3.53	7.38E-05
**positive regulation of cell migration (GO:0030335)**	480	20	5.81	+	3.44	2.39E-02
**regulation of vesicle-mediated transport (GO:0060627)**	509	21	6.16	+	3.41	1.55E-02
**regulation of body fluid levels (GO:0050878)**	485	20	5.87	+	3.41	2.77E-02
**cell activation (GO:0001775)**	1045	43	12.64	+	3.4	3.56E-08
**response to wounding (GO:0009611)**	562	23	6.8	+	3.38	5.60E-03
**secretion by cell (GO:0032940)**	978	40	11.83	+	3.38	2.66E-07
**response to cytokine (GO:0034097)**	1028	42	12.44	+	3.38	8.18E-08
**cellular response to cytokine stimulus (GO:0071345)**	941	38	11.38	+	3.34	1.26E-06
**regulation of cell motility (GO:2000145)**	875	35	10.59	+	3.31	9.26E-06

**positive regulation of cell motility (GO:2000147)**	501	20	6.06	+	3.3	4.42E-02
**response to other organism (GO:0051707)**	952	38	11.52	+	3.3	1.73E-06
**response to external biotic stimulus (GO:0043207)**	954	38	11.54	+	3.29	1.83E-06
**cell adhesion (GO:0007155)**	915	36	11.07	+	3.25	7.98E-06
**positive regulation of locomotion (GO:0040017)**	534	21	6.46	+	3.25	3.19E-02
**activation of immune response (GO:0002253)**	560	22	6.77	+	3.25	1.88E-02
**chemotaxis (GO:0006935)**	536	21	6.48	+	3.24	3.37E-02
**biological adhesion (GO:0022610)**	921	36	11.14	+	3.23	9.44E-06
**taxis (GO:0042330)**	538	21	6.51	+	3.23	3.56E-02
**cytokine-mediated signaling pathway (GO:0019221)**	615	24	7.44	+	3.23	7.14E-03
**response to biotic stimulus (GO:0009607)**	980	38	11.86	+	3.21	3.81E-06
**regulation of secretion (GO:0051046)**	784	30	9.48	+	3.16	4.15E-04
**regulation of locomotion (GO:0040012)**	951	36	11.5	+	3.13	2.14E-05
**response to lipid (GO:0033993)**	848	32	10.26	+	3.12	1.95E-04
**secretion (GO:0046903)**	1092	41	13.21	+	3.1	1.80E-06
**immune system development (GO:0002520)**	628	23	7.6	+	3.03	3.40E-02

**regulation of cellular component movement (GO:0051270)**	956	35	11.57	+	3.03	8.32E-05
**regulation of response to stress (GO:0080134)**	1341	49	16.22	+	3.02	5.69E-08
**immune response (GO:0006955)**	1816	65	21.97	+	2.96	1.97E-11
**cell migration (GO:0016477)**	930	33	11.25	+	2.93	4.70E-04
**regulation of MAPK cascade (GO:0043408)**	737	26	8.92	+	2.92	1.50E-02
**regulation of secretion by cell (GO:1903530)**	726	25	8.78	+	2.85	3.62E-02
**anatomical structure formation involved in morphogenesis (GO:0048646)**	877	30	10.61	+	2.83	4.23E-03
**positive regulation of intracellular signal transduction (GO:1902533)**	1054	36	12.75	+	2.82	2.78E-04
**regulation of cell population proliferation (GO:0042127)**	1596	54	19.31	+	2.8	6.82E-08
**positive regulation of phosphorylation (GO:0042327)**	1031	34	12.47	+	2.73	1.56E-03
**immune system process (GO:0002376)**	2687	88	32.51	+	2.71	6.55E-15
**positive regulation of phosphorus metabolic process (GO:0010562)**	1103	36	13.34	+	2.7	8.31E-04
**positive regulation of phosphate metabolic process (GO:0045937)**	1103	36	13.34	+	2.7	8.31E-04

**positive regulation of cell differentiation (GO:0045597)**	951	31	11.5	+		2.69	7.27E-03
**negative regulation of multicellular organismal process (GO:0051241)**	1167	38	14.12	+		2.69	3.70E-04
**negative regulation of programmed cell death (GO:0043069)**	895	29	10.83	+		2.68	1.90E-02
**positive regulation of multicellular organismal process (GO:0051240)**	1690	54	20.44	+		2.64	5.57E-07
**negative regulation of apoptotic process (GO:0043066)**	880	28	10.65	+		2.63	4.02E-02
**localization of cell (GO:0051674)**	1038	33	12.56	+		2.63	5.34E-03
**cell motility (GO:0048870)**	1038	33	12.56	+		2.63	5.34E-03
**negative regulation of cell death (GO:0060548)**	977	31	11.82	+		2.62	1.26E-02
**positive regulation of protein phosphorylation (GO:0001934)**	980	31	11.86	+		2.61	1.34E-02
**response to external stimulus (GO:0009605)**	2062	65	24.95	+		2.61	6.19E-09
**negative regulation of developmental process (GO:0051093)**	923	29	11.17	+		2.6	3.40E-02
**positive regulation of cell population proliferation (GO:0008284)**	924	29	11.18	+		2.59	3.47E-02

**regulation of programmed cell death (GO:0043067)**	1523	47	18.42	+	2.55	3.67E-05
**regulation of protein phosphorylation (GO:0001932)**	1401	43	16.95	+	2.54	3.55E-04
**regulation of apoptotic process (GO:0042981)**	1508	46	18.24	+	2.52	1.11E-04
**positive regulation of molecular function (GO:0044093)**	1764	53	21.34	+	2.48	8.56E-06
**regulation of cell death (GO:0010941)**	1645	49	19.9	+	2.46	5.45E-05
**regulation of phosphorylation (GO:0042325)**	1517	45	18.35	+	2.45	2.92E-04
**positive regulation of response to stimulus (GO:0048584)**	2320	68	28.07	+	2.42	5.83E-08
**positive regulation of protein modification process (GO:0031401)**	1197	35	14.48	+	2.42	1.73E-02
**negative regulation of response to stimulus (GO:0048585)**	1540	45	18.63	+	2.42	4.26E-04
**locomotion (GO:0040011)**	1270	37	15.36	+	2.41	8.90E-03
**regulation of intracellular signal transduction (GO:1902531)**	1844	53	22.31	+	2.38	4.35E-05
**positive regulation of signal transduction (GO:0009967)**	1581	45	19.13	+	2.35	1.13E-03
**positive regulation of developmental**	1340	38	16.21	+	2.34	1.67E-02

**process (GO:0051094)**						
**positive regulation of signaling (GO:0023056)**	1744	49	21.1	+	2.32	3.37E-04
**positive regulation of cellular protein metabolic process (GO:0032270)**	1553	43	18.79	+	2.29	3.99E-03
**regulation of localization (GO:0032879)**	2670	73	32.3	+	2.26	1.72E-07
**positive regulation of cell communication (GO:0010647)**	1736	47	21	+	2.24	2.26E-03
**cellular response to chemical stimulus (GO:0070887)**	2760	74	33.39	+	2.22	3.51E-07
**regulation of phosphate metabolic process (GO:0019220)**	1716	46	20.76	+	2.22	3.86E-03
**tissue development (GO:0009888)**	1717	46	20.77	+	2.21	3.90E-03
**regulation of phosphorus metabolic process (GO:0051174)**	1718	46	20.78	+	2.21	3.95E-03
**regulation of transport (GO:0051049)**	1803	48	21.81	+	2.2	2.30E-03
**regulation of multicellular organismal process (GO:0051239)**	2981	79	36.06	+	2.19	7.01E-08
**response to stress (GO:0006950)**	3412	90	41.28	+	2.18	1.48E-09
**regulation of protein modification process (GO:0031399)**	1792	47	21.68	+	2.17	6.44E-03

**regulation of catalytic activity (GO:0050790)**	2306	60	27.9	+	2.15	1.30E-04
**positive regulation of protein metabolic process (GO:0051247)**	1655	43	20.02	+	2.15	2.89E-02
**cellular response to organic substance (GO:0071310)**	2252	58	27.24	+	2.13	3.24E-04
**regulation of cellular protein metabolic process (GO:0032268)**	2550	65	30.85	+	2.11	5.53E-05
**anatomical structure morphogenesis (GO:0009653)**	2093	53	25.32	+	2.09	2.46E-03
**response to organic substance (GO:0010033)**	2869	72	34.71	+	2.07	1.06E-05
**regulation of response to stimulus (GO:0048583)**	4232	106	51.2	+	2.07	8.44E-11
**cell surface receptor signaling pathway (GO:0007166)**	2374	59	28.72	+	2.05	7.59E-04
**regulation of molecular function (GO:0065009)**	3222	80	38.98	+	2.05	1.26E-06
**vesicle-mediated transport (GO:0016192)**	1910	47	23.11	+	2.03	2.82E-02
**positive regulation of cellular metabolic process (GO:0031325)**	3246	79	39.27	+	2.01	4.66E-06
**regulation of protein metabolic process (GO:0051246)**	2726	66	32.98	+	2	2.87E-04
**positive regulation of metabolic process (GO:0009893)**	3534	83	42.75	+	1.94	1.01E-05

**multi-organism process (GO:0051704)**	2429	57	29.38	+	1.94	9.05E-03
**regulation of developmental process (GO:0050793)**	2486	58	30.07	+	1.93	1.09E-02
**positive regulation of nitrogen compound metabolic process (GO:0051173)**	3128	72	37.84	+	1.9	3.98E-04
**positive regulation of macromolecule metabolic process (GO:0010604)**	3270	75	39.56	+	1.9	2.40E-04
**regulation of signaling (GO:0023051)**	3542	81	42.85	+	1.89	5.43E-05
**regulation of signal transduction (GO:0009966)**	3158	71	38.2	+	1.86	1.28E-03
**regulation of cell communication (GO:0010646)**	3514	78	42.51	+	1.83	4.02E-04
**response to chemical (GO:0042221)**	4331	96	52.39	+	1.83	3.83E-06
**positive regulation of cellular process (GO:0048522)**	5280	116	63.88	+	1.82	1.88E-08
**positive regulation of biological process (GO:0048518)**	6000	131	72.59	+	1.8	2.18E-10
**cell differentiation (GO:0030154)**	3619	74	43.78	+	1.69	3.14E-02
**signal transduction (GO:0007165)**	4898	100	59.25	+	1.69	1.37E-04
**cellular developmental process (GO:0048869)**	3711	75	44.89	+	1.67	3.95E-02

**signaling (GO:0023052)**	5215	105	63.09	+	1.66	1.14E-04
**cell communication (GO:0007154)**	5335	107	64.54	+	1.66	9.52E-05
**cellular response to stimulus (GO:0051716)**	6484	129	78.44	+	1.64	6.59E-07
**regulation of biological quality (GO:0065008)**	3924	78	47.47	+	1.64	4.32E-02
**response to stimulus (GO:0050896)**	8293	163	100.32	+	1.62	4.09E-11
**negative regulation of biological process (GO:0048519)**	5148	98	62.28	+	1.57	6.79E-03
**regulation of biological process (GO:0050789)**	11531	183	139.5	+	1.31	3.12E-04
**regulation of cellular process (GO:0050794)**	10812	170	130.8	+	1.3	8.41E-03
**biological regulation (GO:0065007)**	12220	191	147.83	+	1.29	2.32E-04
**biological_process (GO:0008150)**	17746	239	214.68	+	1.11	3.72E-02
**Unclassified (UNCLASSIFIED)**	3250	15	39.32	-	0.38	0.00E+00
